# Titanium in Cast Cu-Sn Alloys—A Review

**DOI:** 10.3390/ma14164587

**Published:** 2021-08-16

**Authors:** Karthik Manu, Jan Jezierski, Madikkamadom Radhakrishnan Sai Ganesh, Karthik Venkitaraman Shankar, Sudarsanan Aswath Narayanan

**Affiliations:** 1 Department of Mechanical Engineering, Amrita Vishwa Vidyapeetham, Amritapuri 690525, India; karthikm74125@gmail.com (K.M.); saig852000@gmail.com (M.R.S.G.); aswath610@gmail.com (S.A.N.); 2 Department of Materials Science and Engineering, KTH Royal Institute of Technology, SE-100 44 Stockholm, Sweden; 3 Department of Foundry Engineering, Silesian University of Technology, Towarowa 7, 44-100 Gliwice, Poland

**Keywords:** creep, intermetallic, cast Cu-Sn, spinodal bronze, cast Cu-Sn-Ti, two-phase zone continuous casting

## Abstract

The article reviews the progress made on bronze alloys processed through various casting techniques, and focuses on enhancements in the microstructural characteristics, hardness, tensile properties, and tribological behaviour of Cu-Sn and Cu-Sn-Ti alloys. Copper and its alloys have found several applications in the fields of automobiles, marine and machine tools specifically for propellers in submarines, bearings, and bushings. It has also been reported that bronze alloys are especially used as an anti-wear and friction-reducing material to make high performance bearings for roller cone cock bits and warships for defence purposes. In these applications, properties like tensile strength, yield strength, fatigue strength, elongation, hardness, impact strength, wear resistance, and corrosion resistance are very important; however, these bronze alloys possess only moderate hardness, which results in low wear resistance, thereby limiting the application of these alloys in the automobile industry. The major factor that influences the properties of bronze alloys is the microstructure. Morphological changes in these bronze alloys are achieved through different manufacturing techniques, such as casting, heat treatment, and alloy addition, which enhance the mechanical, tribological, and corrosion characteristics. Alloying of Ti to cast Cu-Sn is very effective in changing the microstructure of bronze alloys. Reinforcing the bronze matrix with several ceramic particles and surface modifications also improves the properties of bronze alloys. The present article reviews the techniques involved in changing the microstructure and enhancing the mechanical and tribological behaviours of cast Cu-Sn and Cu-Sn-Ti alloys. Moreover, this article also reviews the industrial applications and future scope of these cast alloys in the automobile and marine industries.

## 1. Introduction

Understanding the different properties of an alloy is crucial if its service is to be extended under particular working conditions. Various studies on Cu-Sn alloys have been performed to expand their applications. Lead-based solders used to be very common, but there was a search for a better lead replacement due to its highly toxic nature. Cu-Sn alloys turned out to be a better replacement because of their non-toxic nature and significant abundance. Bronze alloys were found to be a viable material to make primer and detonating cords because of their high impulse transfer. The high conductivity of bronze alloys make them useful as a conducting material for winding applications, which will be described later. Likewise, many properties of Cu-Sn alloy have shown a wide range of compatibility with steel. Due to the high strength and low density of titanium, when compared to other alloying additives, the effects of titanium on various alloys have been studied by many researchers, such as in steel [1]. The paper presents changes in the impact strength and abrasive wear resistance of cast high manganese steel due to the formation of primary titanium carbides. For high chromium cast iron, another group of authors investigated the influence of titanium on its crystallization process and wear resistance [2]. Titanium also possesses the property of corrosion resistance when in contact with seawater or chlorine. This explains its use in marine industries and ships. Furthermore, the Cu-Sn-Ti alloy group has been used to make high-strength ageing materials and abrasive binders. This system showed a lower melting point and good wear properties. The outstanding wetting property of this system was attributed to the strong joints existing between abrasives and matrix. Titanium alloys have also found broad application in biomaterials because of their almost neutral effect on the human body.

The current paper reviews cast Cu-Sn-Ti alloys and presents a wide range of experiments that have been conducted and lists their applications, together with an analysis of bronze alloys and their applications. The superior nature of Cu-Sn-Ti alloys is proved along with an explanation of opportunities for future development and research.

## 2. Cast Bronze Alloys

Cast bronze alloys are broadly identified as C80000 and C90000 series, and are mainly produced by sand casting, and continuous or centrifugal casting process routes. The main intermetallic phases formed during processing include Cu_3_Sn and Cu_6_Sn_5_. Deformation is based on dynamic recrystallization (DRX), where Cu_6_Sn_5_ particles dominate the process of particle stimulated nucleation (PSN) over those triggered by a boundary (boundary induced nucleation). Adding Sn to Cu improves various properties and allows the resulting alloy (bronze) to meet the requirements of many applications. To prove its use as a solar-reflecting substance, Alex et al. [3] researched the behaviour of the Cu-Sn intermetallic system. In this analysis, a customized replacement of aluminium or zinc in the bronze alloy was performed. A single-phase cast bulk alloy (Cu-21.2 wt%Sn) allowed in arc melting and chill casting of bronze alloys, which displayed a solar reflectance of 74.5% and a specular reflectance of 77.2%, combined with a hardness of 5 GPa. In addition, an aluminium substitution of about 4–21.2% was found to increase the solar reflectance of bulk alloy by 10% and the specular reflectance by 14%. A zinc substitution of 2–9%, on the other hand, induced a 1% decrease in bulk specular reflectance and a 1% decrease in bulk solar reflectance. Therefore, it has been shown that traditional metallurgical processing, when compared to multilayer solar-reflective coatings, can produce bulk materials with acceptable solar and specular reflectance. The next section of this analysis focuses on the effects of modifications to Sn and their effects on the microstructure.

## 3. Effect of Sn Additions

**Figure 1:**
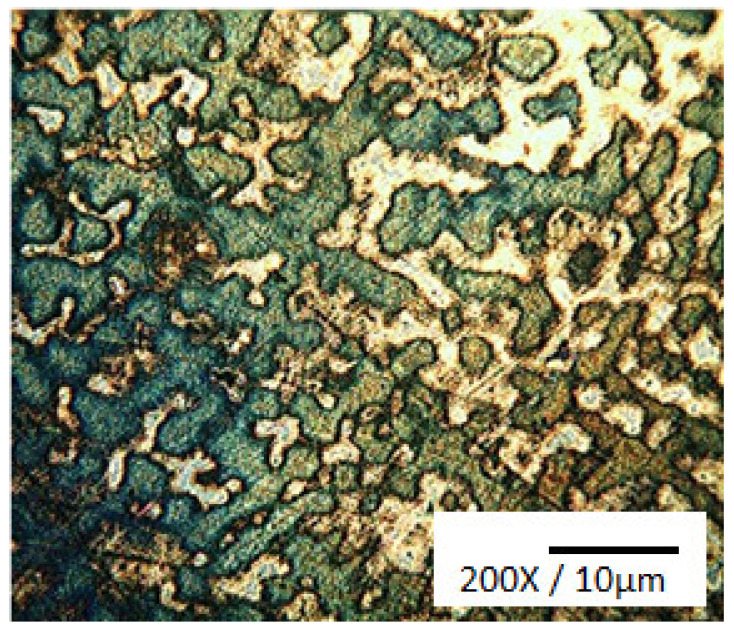
Microstructure of Cu-10Sn alloy in as-cast condition [5].

**Figure 2:**
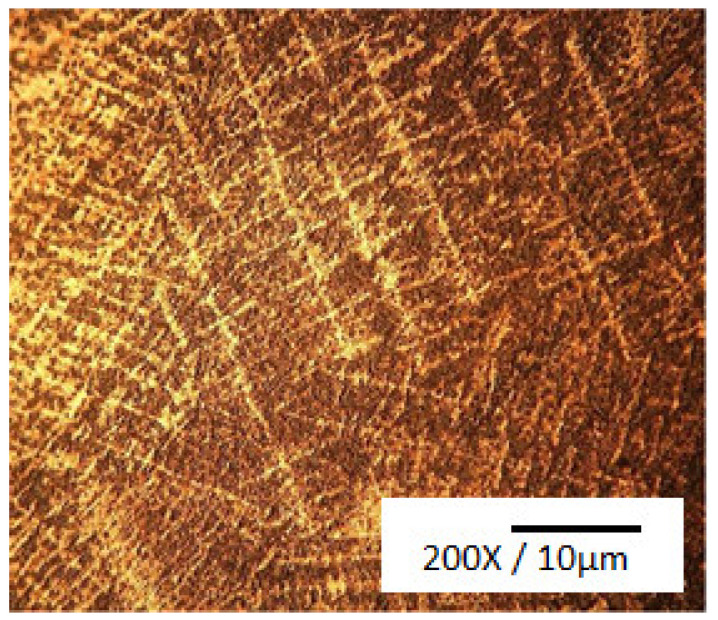
Microstructure of Cu-10Sn alloy in a refined state [5].

**Figure 3:**
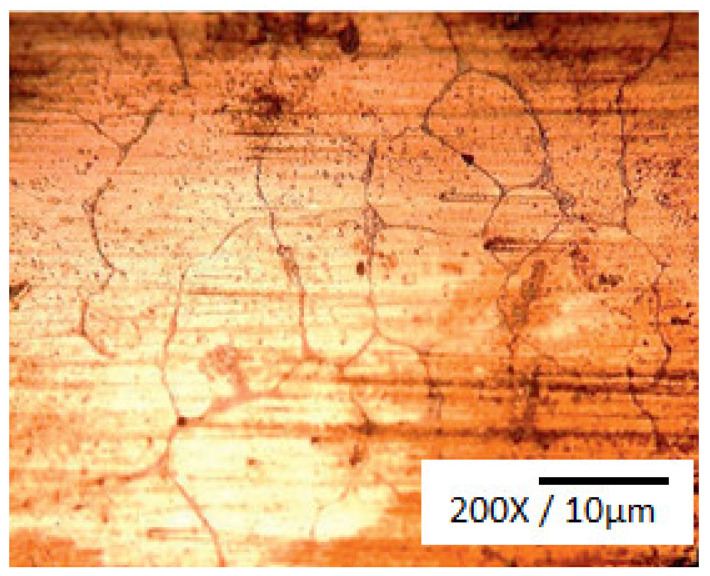
Microstructure of homogenized Cu-10Sn alloy [5].

Because of their high corrosion resistance, as well as their outstanding thermal and electrical conductivities, Cu and its alloys are commonly used in many fields as bearings and bush sleeves, and in many other applications. An excellent way to obtain high strength and fine grains for an alloy is to use the process of severe plastic deformation (SPD). ECAP, or equal channel angular pressing, is a form of material processing that applies very high strains, leading to severe work hardening and the refining of product microstructure. Elshafey et al. [4] clarified the impact of equal channel angular pressing (ECAP) passes, a material processing method that causes very high strains in samples, on the material properties of Cu-Sn alloys. A microstructural refinement that contributes to work hardening is triggered by ECAP. The alloy was processed via ECAP for 1, 4, and 5 passes to a diameter of 12 mm at room temperature, with a 120° inner angle, and a 20° die-out arc angle. It was found that, with an increase in Sn content, or with the number of ECAP passes, the grain size decreased. It was noticed that, for 10% alloys, the proof strength, ultimate tensile strength (UTS), and Cu-2%, 5% and 10% Sn alloy hardness improved with either an improvement in the number of ECAP passes or an increase in the content of Sn. UTS reached its peak after 4 passes for 2% Sn content, and UTS only reached its limit after 3 passes for 5% Sn content. The wear resistance of bronze alloys was also found to be substantially improved by adding more Sn or by increasing the ECAP pass number. A substantial decrease in surface roughness could also be achieved by increasing the number of ECAP passes or by decreasing the content of Sn. In addition, the effects of Sn addition on the microstructure, wear properties, and surface hardness of Cu-Sn refined alloys [5] was investigated; gas tungsten arc was used in the study as the heat source to conduct surface refining. Bronze alloys with various compositions of Sn were chosen for the test. Using an optical microscope, a microstructural examination was carried out, and hardness was assessed using the Vickers hardness test. As shown in Figure 1, the Cu-10Sn alloy microstructural investigation of the as-cast condition led to confirm the occurrence of a dendritic structure. The Cu-10Sn alloy surface refining, as shown in Figure 2, resulted in a microstructure with fine grains. This shows that, by conducting surface refining, the hardness and wear properties of Cu-10Sn alloys can be improved. Homogenization of the refined Cu-10Sn alloy surface led to a microstructure without dendrites as shown in Figure 3. In addition, observations under optical microscopes showed that, even after providing alloy heat treatment, the second step did not occur. The hardness and wear properties were found to be dependent on the Sn content. The relationship between hardness and wear rate was found to comply with Archard’s theory. The wear rate was found to decrease with Sn addition. In addition, it was found that the coefficient of friction was constant and independent of hardness.

**Figure 4:**
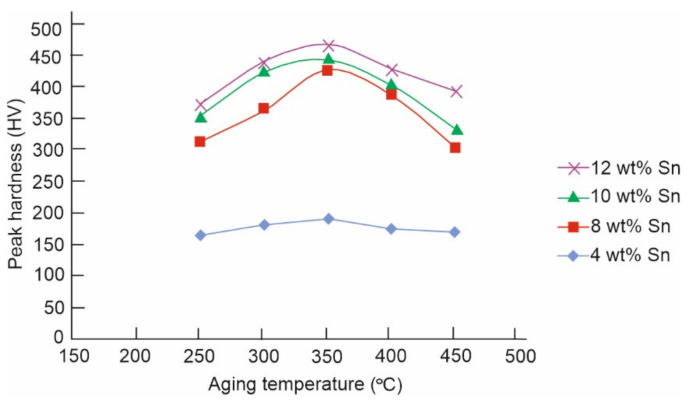
Peak hardness versus ageing temperature [6].

To obtain the variance of hardness and wear properties for Cu-10Sn alloys, an experiment was performed. Minor additions of nickel were made to bronze alloys because bronze alloys showed a low tensile strength. This resulted in obtaining spinodal alloys with excellent corrosion resistance, fatigue property, and tensile strength. Therefore, to govern the effect of Sn addition on the microstructure and hardness of spinodal bronze alloys, an experiment was performed by Shankar et al. [6]. Three of the elements were combined by heating them in an electric furnace at a temperature of 825 °C for 10 h and then aged for 1–5 h at 250, 300, 350, 400, and 450 °C. Experimental results were obtained in the form of a plot between peak hardness and ageing temperature. Figure 4 shows that with an increase in the content of Sn from 4–12 wt%, there was an improvement in the peak hardness value. In addition, the peak ageing period was independent of the Sn content. It was found that the optimal ageing period and optimal ageing temperature were 3 h and 350 °C, respectively. When the alloy was over-aged, this analysis additionally confirmed the formation of grain boundary precipitates.

## 4. Properties of Bronze Alloys

**Figure 5:**
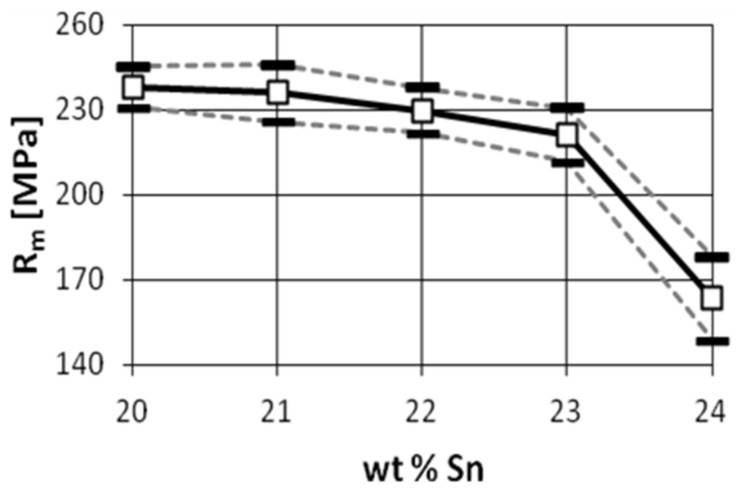
Tensile strength of high-tin bronzes versus tin content [8].

**Figure 6:**
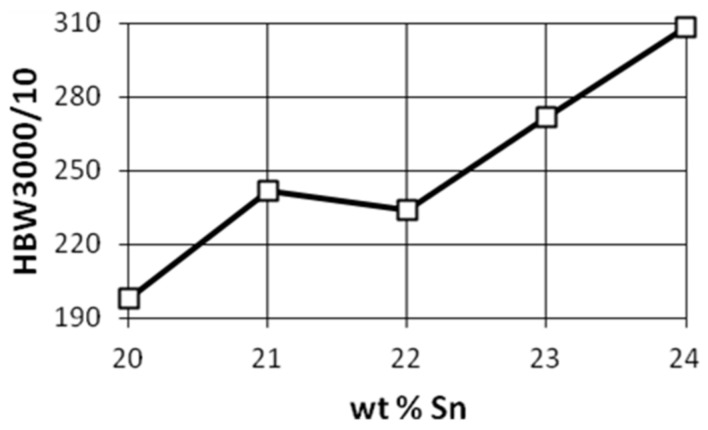
The hardness of high-tin bronzes versus tin content [8].

**Figure 7:**
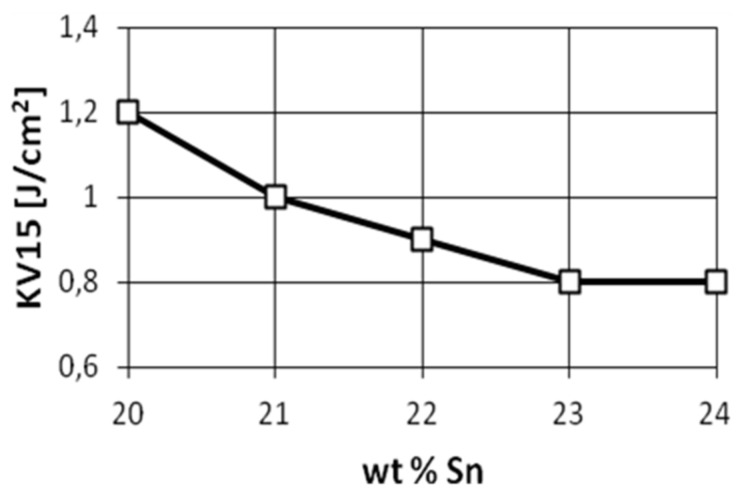
Impact strength of high-tin bronzes versus tin content [8].

Owing to their high strength, good wear resistance, good corrosion resistance, and simple casting properties, Cu-Sn alloy has been widely used in many fields. Rapid solidification processing is an alloy preparation process that, unlike ordinary processes, can result in a polished microstructure and produce a material with superior efficiency. Various properties and production methods have been studied to enhance the properties of the alloys. As presented in [7], solidification rate has a significant impact on a high-tin bronze microstructure. Other research projects presented how tin content changes the mechanical properties of bronzes [8]. Figure 5, Figure 6 and Figure 7 show the tensile strength, hardness, and impact strength for various tin contents.

**Figure 8:**
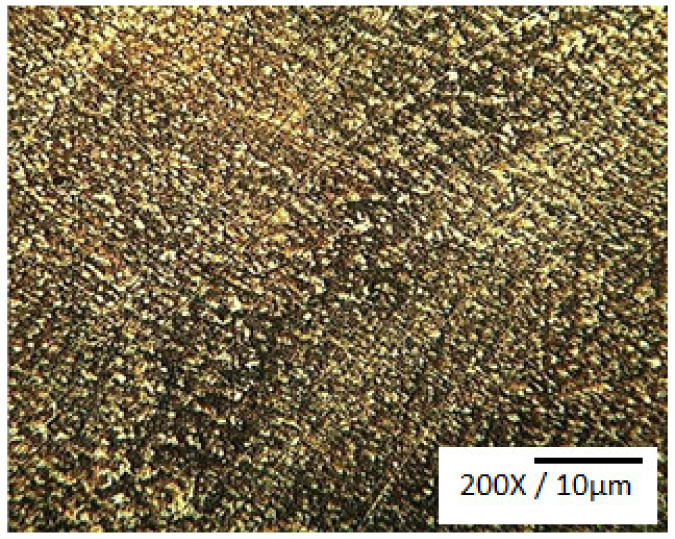
Modified structure of Cu-10Sn alloy [9].

The effects of the process parameters on surface-modified Cu-Sn alloy microstructure, hardness, and wear properties were investigated by Cherian et al. [9]. Their surface modification method used a tungsten gas arc serving as the source of heat. A pin-on-disc wear tester and a micro-hardness tester were used to determine the wear rate and layer hardness. It was found that, when compared to as-cast samples, the surface modification process enhanced the alloy hardness and wear properties. In addition, as shown in Figure 8, the presence of uniformly distributed fine grains was confirmed in the microstructure of the modified layer. Hence, a refined microstructure was provided by bronze alloy surface modification. The wear rate was found to decrease with hardness, and the coefficient of friction was found to be independent of hardness.

**Figure 9:**
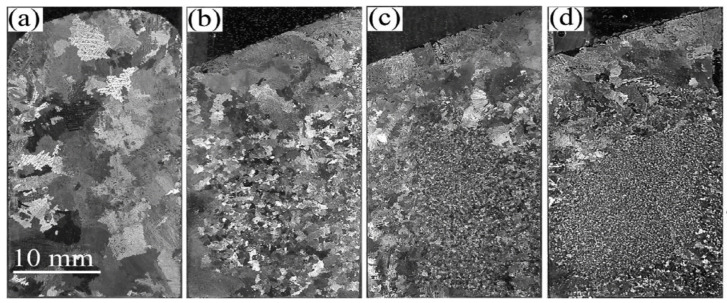
Cu-11 wt%Sn macrostructure at (**a**) G = 1, (**b**) G = 100, (**c**) G = 300, and (**d**) G = 600 [12].

Zhang et al. [10] investigated the resistivity and mechanical properties of various compositions of bronze alloys. Bronze (Cu-0.5 wt% Sn) alloys were isothermally annealed after cold rolling and found to have 136.95% of the International Annealed Copper Standard (IACS) electrical conductivity. With the addition of Sn, electrical conductivity and mechanical elongation decreased. On the other hand, the addition of Sn provided a substantial increase in the alloy’s tensile strength. It was also concluded that the annealing procedure improved alloy conductivity and mechanical elongation. In studies conducted by Salmat et al. [11], the effect of a high-tin bronze chemical composition on the physical, mechanical, and acoustic properties of gamelan materials was investigated. Yang et al. [12] investigated the effect of the super-gravity field on Cu-Sn alloy tensile properties and grain refinement. In their analysis, two furnaces were used in the apparatus, rotating around a fixed axis. It was concluded that Cu-11 wt% Sn alloy grain refinement was promisingly higher in a super-gravity region. The average grain size was found to be 2.13 mm in the normal gravity field, while it was 0.35 mm, 0.173 mm, and 0.074 mm in the super-gravity fields of G (gravity coefficient) = 100, 300, and 600. The reduction in grain size was confirmed in Figure 9, where there is a greater strength of the super-gravity field. Cu-11 wt% Sn in the normal gravity field achieved a tensile strength value of 265 MPa while 449 MPa, 487 MPa, and 521 MPa were achieved for super-gravity fields of G = 100, 300, and 600, respectively. The morphology of failure changed from fragility to plasticity with raising the gravity coefficient. The collapse of crystal nuclei into the solidified melt was only greatly exacerbated by super-gravity at the early stage of solidification, which contributed to solid structure refinement. An increased concentration of Sn has been found to help grow grain refinement, thus increasing the rate of nucleation. The increase in tensile characteristics can be explained by the grain size reduction.

The microstructure, friction coefficient, surface stiffness, material of particles, thermal conductivity, and weight loss of Fe-Cr-reinforced Cu-Sn metal matrix composites (MMCs) were investigated by Yilmaz et al. [13]. The alloy was tested under a load of 125 N and within a temperature range of 300–475 K. Dry sliding measurements were also carried out in increments of 300 m per increment, for a total length of 3500 m. This research found that Cu-Sn showed lower wear resistance than Cu-Sn MMCs reinforced with FeCr. Wear was found to continue due to the adhesion process. It was also found that, when the surface temperature reached a critical value, the transition to extreme wear (which entails extensive surface degradation and transfer of material to the counter face) took place. Moreover, a relationship was established between the thermal conductivity and friction coefficient (material with a higher thermal conductivity promoted a low friction coefficients). In addition, it was found that material with a higher thermal conductivity was more prone to transition induced by severe wear. A similar relationship was observed for the coefficient of friction (COF) and grain boundary energy. Furthermore, Osorio et al. [14] proved that electrochemical corrosion resistance improves in coarse microstructural arrays when it is directionally solidified. Cooling rates for the solidification of Sn-2.8 wt%Cu alloy were controlled to obtain information regarding segregation pattern, capacitance, Vickers microhardness, and polarization resistance, thereby giving a clear idea of the changes taking place in the microstructure during the solidification process. To proceed with the above-mentioned experiment, there should be accurate results for unidirectional solidification to increase corrosion resistance. Martorano et al. [15] chose Cu-8 wt%Sn to investigate the variation in heat transfer coefficient occurring at the metal–mould interface. Martorano et al. [15] also derived an algorithm to predict the heat transfer coefficient variance by considering experimental temperature curves as the function of time used as algorithm inputs.

**Figure 10:**
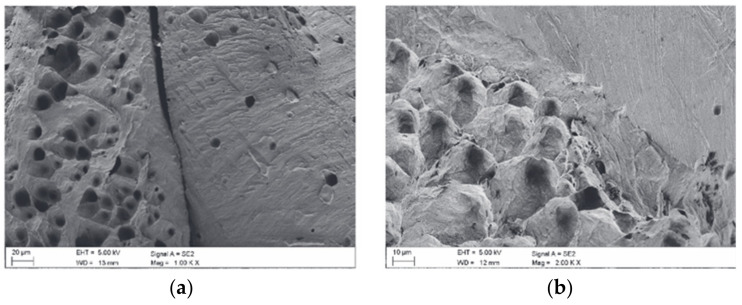
Fracture surface at temperature (**a**) 150 °C; (**b**) 250 °C [16].

Tensile tests on tin bronzes were performed at temperatures ranging from 150 °C to 250 °C [16]. The micrographs, as shown in Figure 10, present the fractures of the samples under examination. The deformation temperature is the main factor in deciding the fractography of measured surfaces. Annealed and continuously cast bronze in the transition temperature range from ductility to brittleness, CuSn6P showed a mixed fracture in the vicinity of smooth cleavage surfaces, as shown in Figure 10a. This was a distinctive brittle inter-crystalline cracking occurrence. It was found that ductile surfaces in the fracture were less frequent with an increase in deformation temperature, as shown in Figure 10b. On the inter-crystalline surfaces, in the TE range, traces of plastic deformation and cavitation were observed. In contrast, deformation happens as there is a change in temperature which lead to fracture formation on fully inter-crystalline brittle surfaces. Inter-crystalline fissures have been discovered to nucleate at grain boundaries at micro-voids, and at the intersections between deformation bands and the grain boundary. It was also found that intercrystalline cracking found between two or three grains on the boundary surfaces ran through cavitation pores, which is typically observed in cast structures.

**Figure 11:**
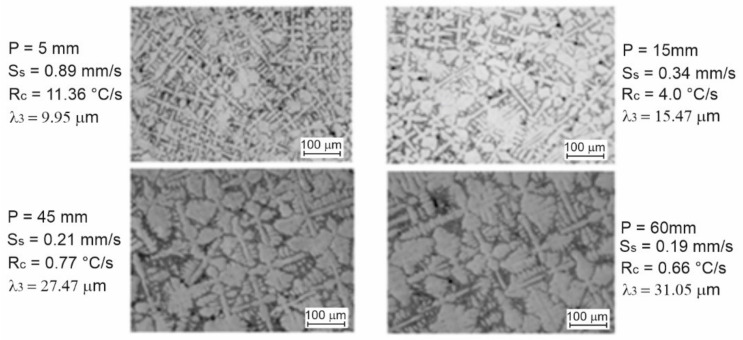
Morphological behaviour of Cu-Sn alloy positioned at 5, 15, 45, and 60 mm [17].

A research project was conducted to determine the effects of thermal factors on the morphological and mechanical behaviour of directionally solidified Cu-Sn alloy [17]. Cu-20 wt% alloy temperature mapping was performed at the time of solidification by fixing thermocouples from a refrigerated base at various positions, namely, 5, 10, 15, 20, 35, 45, and 60 mm. An important improvement in the morphologic activity of the Cu-Sn alloy was observed. It was noted that, when compared to the position of 60 mm, finer dendritic structures were detected at the position of 5 mm. This is because the position of 5 mm was closer to the cooling plate and therefore had a higher cooling rate compared to the position of 60 mm, as shown in Figure 11.

## 5. Analysis of Cu-Sn Cast Samples

The effects of processing factors on the microsegregation of Cu-8 % Sn alloy directionally cast samples was studied [18]. Samples of Cu-Sn alloys were prepared in this study under four experimental conditions, leading to four distinct dendritic growth varieties. By using quantitative metallographic and microprobe analyses, cylindrically formed samples were divided into transverse slices and the degree of microsegregation was observed. The three calculated microsegregation indexes included the segregation variance parameter, volume fraction of the eutectoid, and tin concentration at the centre of the primary dendrite arms. Near the surface of the cast sample, there was a decline in microsegregation. In addition, different levels of microsegregation in the columnar and equiaxial regions of the sample were observed. This was attributed to the difference in inter-dendritic distances. Moreover, this study was able to predict the microsegregation behaviour of peritectic alloys. Bayle et al. [19] experimented with flow stress and recrystallization parameters when Cu-9%Sn alloys (Cu–9Sn and Cu–9Sn–0·26Zn (wt%) were hot deformed from ambient temperature to 750 °C, with nominal (initial) strain rates ranging from 10^−3^ to 10^−1^ s. A plot between true stress and strain curves was determined. Optical metallography was used to determine the dynamic grain recrystallization (DRX). The dynamic recrystallization of grains stimulated a necklace-type structure when exposed to high temperatures. In addition, electron back scattered diffraction (EBSD) measurements were carried out and, as a result, the crucial role of twinning on these alloys was revealed. The heat strain of these alloys has led to improvements in the microstructure and stress–strain curves. Dynamic recrystallization is a slow process and is incompletely propagated when the strain magnitude is 1.2. It was also inferred that solute drag can control the behaviour of a metal alloy. The characteristics of flow curves, such as high-rate sensitivities, lack of work hardening, and traditional transient flow curves, have confirmed this. Twinning leads to recrystallized necklaces being formed along the borders of deformed grains. The electromigration phenomenon was tested by Chen et al. [20] for interfacial reactions in cast Cu-Sn joints. Samples were reacted at 170 °C and 180 °C for 24–240 h by passing an electric current with a density of 5000 A/cm^2^ to perceive the effects of electromigration. At the interface, uniform layers of Cu_6_Sn_5_ and Cu_3_Sn were formed. However, the interfacial reactions were found to be the same, even when an electric current was not supplied, i.e., the electron flows from the Sn side to Cu side. Dissolution of Cu in the Sn matrix during casting initiated a thick Cu_6_Sn_5_ phase. Compared with a previous study, it was found that electromigration played an important role when the direction of the electric current was from the Cu site to the Sn site. Diffusion of Cu near grain boundaries and surfaces promoted the growth of a large non-planar Cu_6_Sn_5_ phase in the microstructure.

Akbarifar et al. [21] compounded cast molten Al melt around a brass cylinder at 700 °C and 750 °C, with 3 and 5 as the melt–solid volume ratios. Upon increasing the temperature and melt–solid volume ratios, the rate of reaction was increased and there were three intermetallic layers. Intermetallic layers consisted of Cu-Zn, Al_4_Cu_3_Zn, and Al_2_Cu along with eutectic α-Al/Al_2_Cu and Al dendrites. The presence of lead-rich phases was confirmed in all layers. XRD and SEM analyses proved the existence of pores at the micro-level and cavities in the dendritic and eutectic layers. The presence of micropores was due to bubble entrapment. There was an indication of a solid ring around the solid core when the brass cylinder rod was cut and observed with SEM. The presence of Al_2_Cu and Al4Cu3Zn layers prevented the saturation of the solid solution with Cu, which resulted in the absence of Cu_4_Al_4_. Moreover, the hardness of the intermetallic layers from the centre of the brass core to the Al outer ring was found to be 513, 477, and 650 Vickers, respectively.

## 6. Phase Diagram of Cu-Sn Alloys

**Figure 12:**
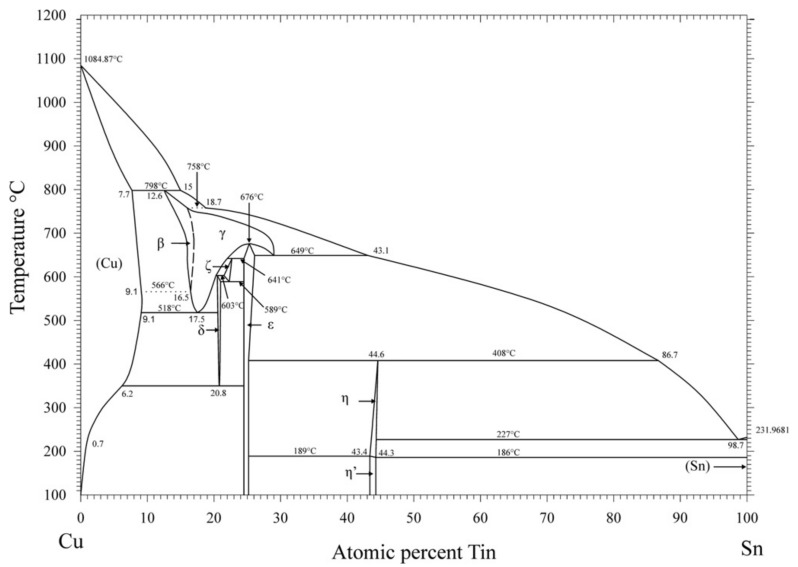
New Cu-Sn phase diagram, as suggested by Furtauer et al. [22].

Since the 19th century, since its introduction, several studies have been conducted to fully understand the Cu-Sn phase diagram and its phase relationships. Binary Cu-Sn alloys are very important as a primary replacement for lead-containing solder materials. By using thermal analysis, metallographic methods, and crystallographic analysis, the alloy phase diagram was determined for samples. As shown in Figure 12, Furtauer et al. [22] implemented a new phase diagram. It was noted that all Sn compositions (from 11% to 48%) were recovered and quenched in cold water, except for the β and γ phases. Since the β (Cu_17_Sn_3_) and γ (Cu_3_Sn) phases were of a high-temperature, they were not quenched, and either transformed into a metastable phase or underwent bulk transformation to their respective low-temperature phases. This β phase can be transformed into the γ phase by a higher-order reaction. Moreover, providing an effective temperature and concentration control facilitates the random orientation of Sn atoms in the BiF_3_ structure. All the results were summed up and expressed in the form of a new phase diagram.

Li et al. [23] investigated the effect of Sn content on the mechanical properties of α-phase Cu-Sn alloys. After casting, it was inferred that the Cu-Sn dendrites were prone to variations in the orientation of the α phase. This was due to a change in the Young’s modulus, which was found to range from 120 to 130 GPa. By incorporating instrumented nano-indentation techniques, this experiment ascertained a linear relationship between the hardness of the α phase and successive additions of Sn.

## 7. Experiments on Molten/Liquid Cu-Sn Alloys

**Figure 13:**
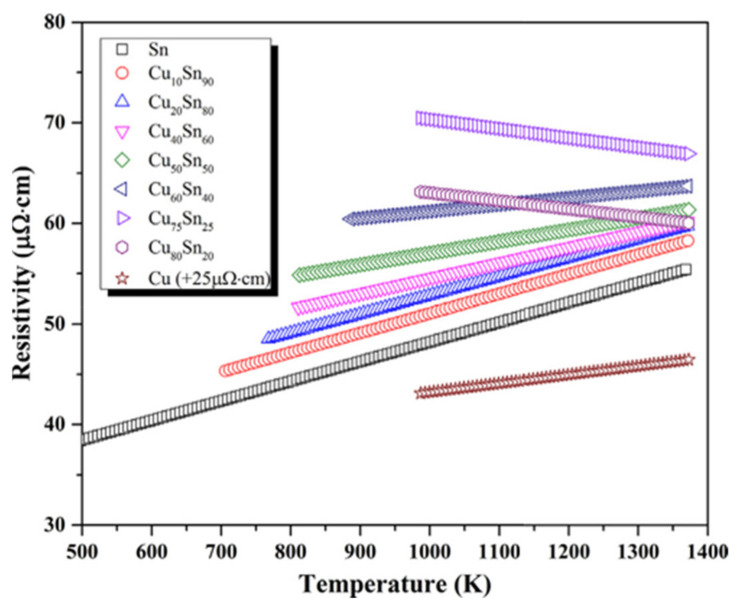
Temperature versus resistivity curve for liquid Cu-Sn alloys with a cooling rate of 5 K/min [24].

The liquid state provided a higher mobility to electrons, which enhanced the conductive property of materials. Liquid structures of bronze alloys can be broadly classified into Cux-Sn100-x where x varies from 0 to 100 (i.e., x = 0, 10, 20, 33, 40, 50, 60, 75, 80, 100). Electrical resistivity and viscosity were the two dominant guiding features in the analysis of transport properties. Jia et al. [24] arrived at three conclusions by examining the transport properties of Cu-Sn alloys. Firstly, when plotting a graph between resistivity and temperature, as shown in Figure 13, Cu_75_Sn_25_ had the lowest value for the temperature coefficient of resistivity (−9.24 μΩcmK^−1^) followed by Cu_80_Sn_20_ (−8 μΩcmK^−1^). Except for these two compositions, all other compositions of Cu-Sn alloys possessed a positive temperature coefficient of resistivity (TCR), which indicated that their resistivity will increase with increasing temperature.

**Figure 14:**
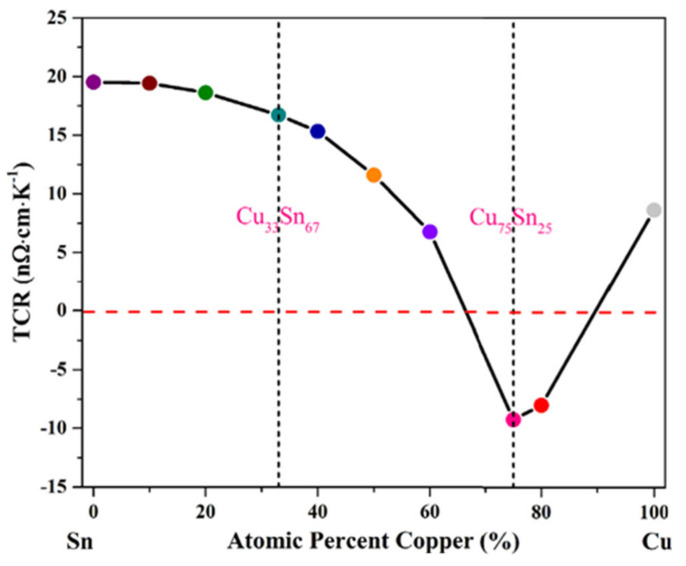
Composition dependence with TCR [24].

Secondly, the variation in resistivity with composition was also studied. Figure 14 shows that an increase in the atomic percentage of copper will result in a decrease in the TCR value until the achievement of a Cu_75_Sn_25_ composition, further increase in Cu content resulted in a lower TCR. This confirmed the presence of atomic clusters in the Cu_75_Sn_25_ alloy with a negative TCR. All the compositions that were similar to this alloy (Cu_75_Sn_25_) possessed a significant number of atomic clusters. Being more different to the composition resulted in a minimal concentration of atomic clusters in the alloy. Hence, Cu_33_Sn_67_ alloys possessed a fine structure.

**Figure 15:**
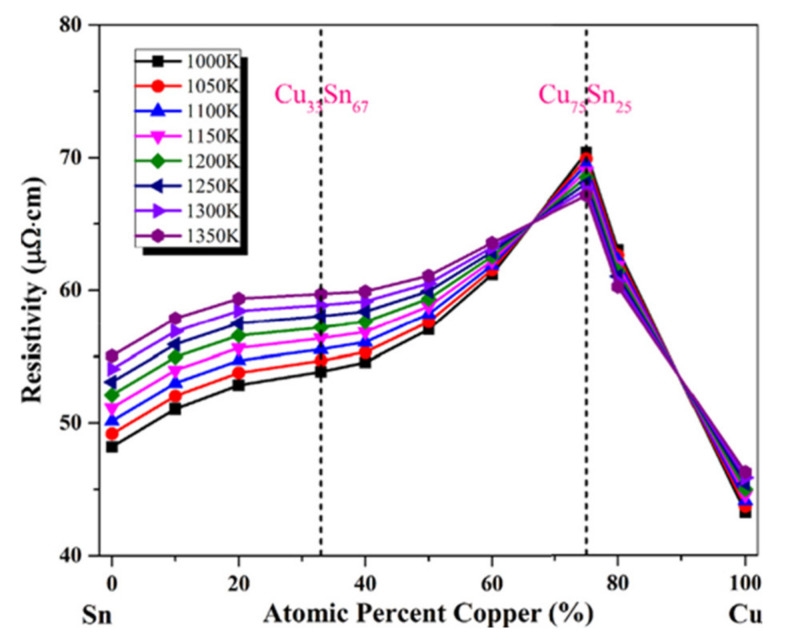
Composition dependent on resistivity for liquid Cu-Sn alloys [24].

Finally, as shown in Figure 15, temperature-dependent resistivity was clarified. In terms of the Arrhenius equation, all compositions of Cu-Sn alloys were found to be have a viscosity that increased with decreasing temperature. Here, Cu_75_Sn_25_ shows a maximum value for the activation energy and the volume of the flow unit. This proved the existence of a stronger bond in Cu_75_Sn_25_ alloy that confirmed the presence of atomic clusters in Cu_75_Sn_25_ alloy. An oscillating-cup viscometer was used by Tan et al. [25] to determine the dynamic viscosities of Cu-Sn alloys at the same superheating temperature. Molten Cu-Sn’s viscosity increased with a decrease in temperature, as found using the Arrhenius equation. It was found that the viscosity was higher near the beta step (Cu5Sn) at a given temperature, along with the peak value occurring at Cu-25 wt% Sn. When Sn concentration was made to range from 20 to 40 wt%, the rate of the viscosity increase with temperature reduction was rapid. In addition, at the same temperature, the viscosity of pure Cu was greater than that of pure Sn alloy. Experiments to observe the effects of a magnetic field on the viscosity of molten Cu-Sn alloys were performed by Mao et al. [26]. An oscillating-cup viscometer was used along with a unique range of Gauss horizontal magnetic fields to obtain the dynamic viscosity of pure Cu and Cu-10% Sn alloy. It was found that, with a large decrease in temperature, the viscosity of Cu-10% Sn increased, while the relationship between temperature and viscosity followed the Arrhenius relationship. With an increase in the power of the magnetic field, the viscosity increased, considering the external magnetic field. When compared to Cu-10% Sn alloy, the effect of the magnetic field on the viscosity of pure Cu was greater.

## 8. Processes Conducted on Cu-Sn Alloy

### 8.1. High-Pressure Torsion

A good grain refinement normally results in SPD or extreme plastic deformation. It also contributes to different phase transitions, such as phase dissolution. To understand the grain refinement induced by Korneva et al. [27], the responses of intermetallic compounds in the Cu-Sn system to SPD were analysed, providing information on the grain refinement of intermetallic compounds when Cu-Sn alloys were subjected to high-pressure torsion (HPT). The sample of Cu-36 wt% Sn chosen for this study consisted of coarse-grained alternating plates of ζ and ε compounds with a thickness of approximately 100 µm. At room temperature, the alloy was exposed to HPT. Increased dislocation density was caused by HPT and led to grain refinement. In the ε process, high-angle grain boundaries were more pronounced. Even after HPT, the macroscopic form of the alternating plates remained unchanged, i.e., the grain refinement took place separately within ζ and ε plates. Although the HPT created cracks in ε phase plates and increased the ε phase hardness, phase transformations and phase dissolutions were absent. Upon completion of HPT, identical conditions were found in the Cu-14 wt% Sn alloy.

### 8.2. Rolling

**Figure 16:**
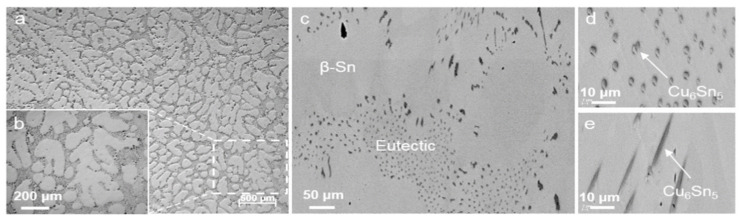
Micrograph and size of primary β-Sn dendrites, eutectic and intermetallic phase in F temper Cu-0.5 wt% alloy when observed under (**a**,**b**) OM, (**c**–**e**) SEM [28].

Cu-Sn alloy can be effectively used as a cladding material in explosives due to its good workability and the property of absorption of small thermal neutrons in a highly radiative environment. Liu et al. [28] incorporated electron backscatter diffraction (EBSD) technique to research the microstructure, dynamic restoration, and grain boundary, twinning, and recrystallization texture of Sn-0.5 wt% Cu alloy. After rolling at a moderate strain rate, the alloy showed a bimodal grain structure comprised of 97% fine grains (less than 30 µm in size) and 3% coarse grains (with 50–150 µm in size). Optical micrograph (OM) and back-scattered SEM micrographs showing the morphology and scale of the as-cast Sn-0.5 wt% Cu alloy in the primary β-Sn, eutectic and intermetallic processes were analysed, as shown in Figure 16. The grain size for the dendritic structure of β-Sn was found to be in the range of 100–300 µm. Both (d) and (e) in Figure 16 confirm the presence of needle-like morphology shapes, 1–3 µm in diameter and 10–20 µm long. In addition, the structure was not present in any other intermetallic phases, such as Cu_3_Sn. Deformation was caused by dynamic recrystallization (DRX), where Cu_6_Sn_5_ particles dominated the process of particle stimulated nucleation (PSN) over those triggered by a boundary (boundary induced nucleation). {3 0 1} and {1 0 1} twins were observed as two additional mechanisms for effective deformation. In addition, the results of the paper by Liu et al. [28] proved that the deformation mechanism initiated by the PSN mechanism leads to the formation of <0 0 1>//RD oriented nuclei. Moreover, a necklace type of structure was also found along the boundary due to continuous dynamic recrystallization (CDRX). It has been observed that <1 1 0>//RD fibre structure is strengthened along with the growth of grains.

**Figure 17:**
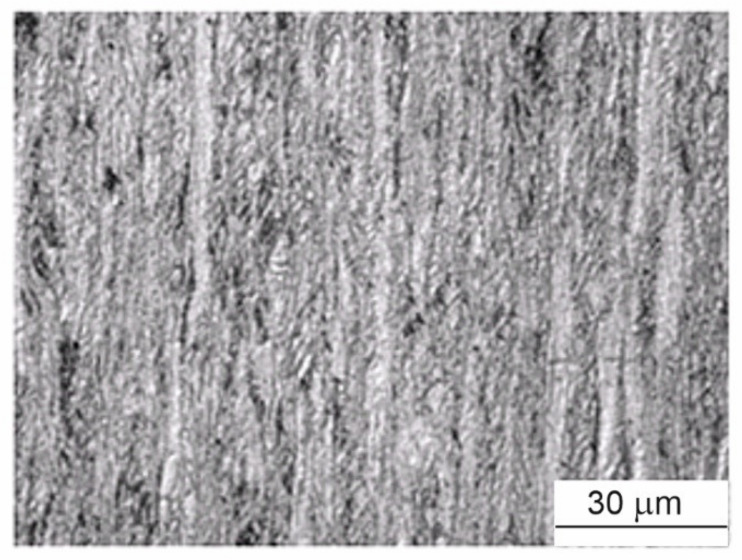
Optical microstructure of cold-rolled Cu-13.5 wt%Sn alloy [29].

In the work of Wang et al. [29], spray forming was used to prepare bronze alloys with high Sn content (Cu-13.5 wt%Sn). In addition, the feasibility and characteristics of cold rolling were investigated. It was found that, as shown in Figure 17, spray-shaped Cu-13.5 wt% Sn had a fine homogeneous single-phase structure, showing excellent workability as compared to standard cast samples. With a 15% decrease in thickness for a single move, the alloy can be cold-rolled, while the overall thickness reduction can reach 80%. The spray formed Cu-13.5 wt%Sn, upon completion of proper thermomechanical treatment, acquired outstanding mechanical properties. Relationships were made between the strength, elongation, and reduction in cross-section. It was also concluded that the resulting alloy acquired a great combination of elastic modulus (88 GPa) and high flow stress (800 MPa) after cold formation. This superior Cu-13.5 wt% Sn alloy property makes them usable in the spring manufacturing industry.

### 8.3. Annealing

Four types of twins were formed at 700 °C in the initial stages of annealing. They were twins on the edge, partial twins, complete twins, and enclosed twins. In the as-cast Cu-4 wt% Sn alloy, no annealing twins were present. In the initial stages of annealing, edge twins were verified, which conformed to the Full man and Fisher model. Enclosed twins were only identified at 700 °C after 40 min of annealing. When the time approached 60 min, at the same annealing temperature, there was a loss of dendritic structures. Two mechanisms were put forth by Liu et al. [30] to explain the formation of twin grains. The first was the tin diffusion mechanism during annealing, i.e., stress fields created were attributed as the reason for recrystallization and twinning induction. Another mechanism that explained the formation of twinned grain was diffusion induced grain boundary migration (DIGM), utilizing the Kirkendall effect.

The tensile properties of bronze alloys were the least affected when annealing temperature ranged from 400 to 750 °C. Han et al. [31] fabricated bronze using a mix-alloy process, i.e., strengthened by dispersion of nano-scale refractory particles. This process seemed to be useful and stable under high-temperature applications. There was a significant improvement in tensile strength when the cold drawing ratio was increased. The increased annealing temperature for cold-drawn dispersion strengthened (DS) bronze decreased the levels of tensile strength and yield strength. This also confirmed that failure was due to the presence of splits in the surface of the DS bronze. This was the main reason for undissolved amounts of Ti and B elements, along with TiB_2_ compounds. DS bronze showed high mechanical properties when compared to commercial phosphorous bronze.

## 9. Casting Processes Conducted on Cu-Sn Alloys

### 9.1. Investment Casting

One of the most used types of metal casting is investment casting. The benefits of this technique include the ability to manufacture goods with thin walls, smooth surfaces, precise shapes, and dimensions. One of the main criteria regulating the consistency of the casting is fluidity. Fluidity is a liquefied molten metal’s ability to glide and fill each part of a mould. Two key parameters, composition and pouring temperature, have a major impact on this property. The main objective of Slamet et al. [32] was to examine the effect of Sn additions on Cu-(20-25) wt%Sn alloy composition and the impact of pouring temperature used in investment casting methods on microstructure and fluidity. The fluidity of Cu-Sn alloys was discovered to improve with an increasing cavity thickness and pouring temperature. A decrease in fluidity would increase the content of Sn. Therefore, the pouring temperature should be increased, and the content of Sn must be reduced to minimize casting defects in Cu-Sn alloys.

### 9.2. Two-Phase Zone Continuous Casting

The correlation between surface quality and process parameters was well established by Liu et al. [33]. A smooth surface of Cu-4.7Sn alloy can be obtained by controlling the speed of continuous casting and the temperature at the entrance of the two-phase zone. With a cooling water temperature of 18 °C, a smooth surface was preserved, and the cooling water flow rate was set to 400 L/h. The microstructure of the resulting alloy showed that Al formed columnar grains near the edge of the alloy plate, grain-covered grains (GCG), continuous columnar, and equiaxed grains in the middle, along with small grains with self-closed-grain boundaries. To preserve the smooth surface properties, the continuous casting speed and temperature were set to 20–30 mm/min and 1020–1040 °C, respectively, at the entrance of the two-phase zone mould.

**Figure 18:**
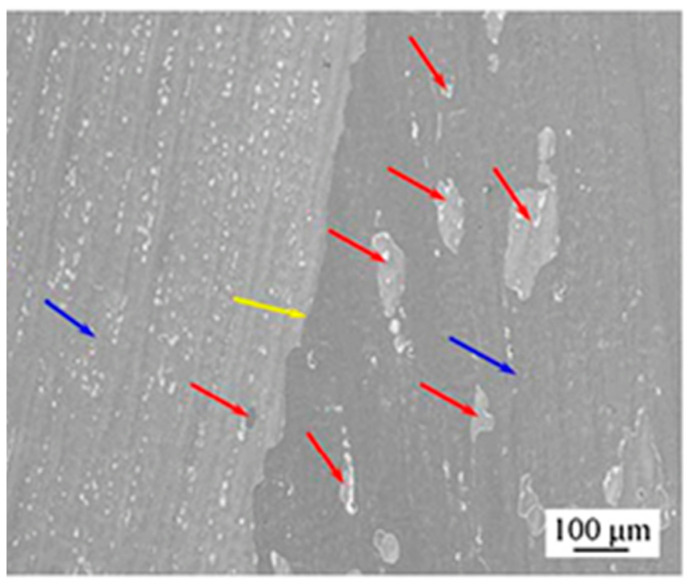
SEM image of GCGs in the microstructure of Cu-Sn alloy after TZCC [34].

Important mechanical properties that govern the failure mechanisms, corrosion resistance, and conductivity of Cu-Sn alloy with a wide range of solid–liquid two-phase zone interfaces were investigated by Liu et al. [34], see Figure 18. This study compared specimens processed using water-cooled mould continuous casting (WMCC) with the specimens produced using two-phase zone continuous casting (TZCC). Covered continuous columnar grains along with non-columnar small grains with self-closed grain boundaries were observed during the TZCC process. They both had the same phase microstructure. Alloys that have undergone TZCC show enhanced ductility and a 255-MPa tensile strength. The tensile strength increase was due to the existence of multiple self-closed grain boundaries that inhibited the motion of dislocation. Moreover, this reason can also be attributed to the excellent corrosion resistance of the alloy, i.e., the grain boundary corrosion was limited. An increase in ductility and a decrease in electrical resistance was due to the presence of continuous columnar grains in the microstructure of the resultant alloy. Conductivity increased to 12.2% when maintained at room temperature. In addition, elongation to failure of the resulting alloy was observed to be at the level of 49%. After TZCC processing of Cu-Sn alloy, large columnar and small grains were obtained. The term grain-covered grains (GCG) was used when small grains were found to be covered with large columnar grains. Five processing parameters (cooling distance, speed of TZCC, the temperature of the melt, mould, and cooling water) were inputted into the back propagation (BP) artificial neural network to obtain the characteristics of their microstructures. From Luo et al. [35], accurate predictions for the microstructure were made using the concept of nine quantities (size of columnar grains, size of small grains, number of columnar grains with small grains, number of columnar grains without small grains, the maximum and minimum number of small grains within columnar grains, number of small columnar grains within columnar grains and boundaries of columnar grains or at the surface of alloy).

**Figure 19:**
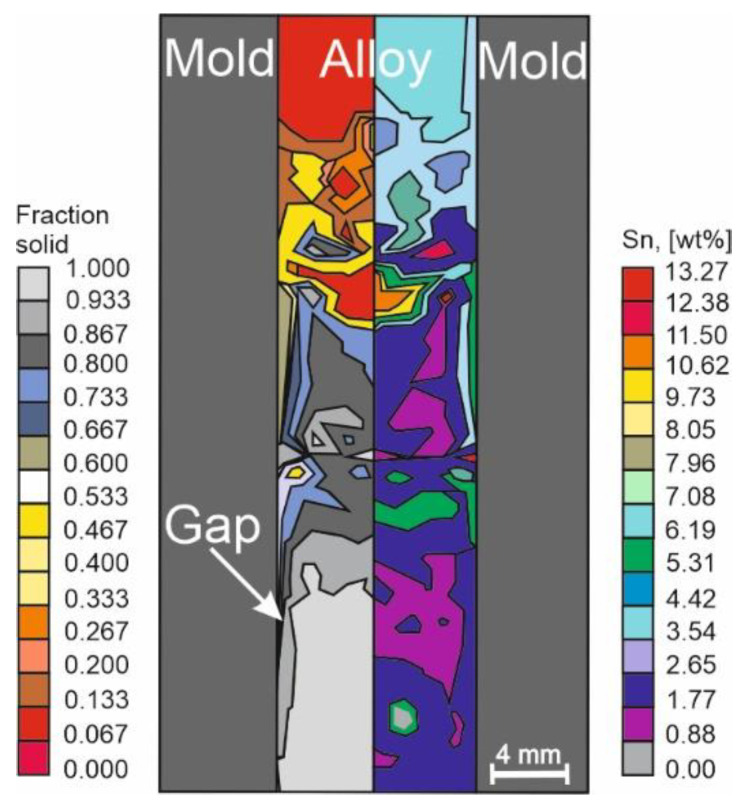
Schematic simulation results of solid–liquid interface morphology and Sn distributio[37].

Microstructural investigation using an optical microscope and the EBSD technique by Luo et al. [36] proved that initial nucleation of small grains resulted in the front of the solid–liquid interface region during TZCC. The angular difference between [0 0 1] columnar grains and the direction of heat flow was found to be less than 19°, which was attributed to the faster growth rate. Similarly, the slower growth rate for small grains was because of the magnitude of the angle exceeding 19° between [0 0 1] small grains and the direction of heat flow. When both columnar and small grains were in contact, the growth of columnar grains was more pronounced when compared to small grains. This led to the formation of GCGs and much smaller grains at the grain boundaries of columnar grains. Microstructure and its quantification have been well-explained by Luo et al. [36] after the TZCC process. Finally, Luo et al. [37] made use of ProCast software that simulates the TZCC process to analyse solute (Sn) distribution and surface segregation taking place along the process. Cu-4.7 wt%Sn alloy with a broader solid–liquid phase zone was under investigation. Figure 19 shows that the solidification of the alloy was first found at the centre and initiated the ‘Λ’ structure. Moreover, a narrow gap was formed between the solid/liquid interface and a wall in the two-phase zone mould was created. This formation of the gap was attributed to the formation of the ‘Λ’ structure. The alloy solidified along the direction opposite to the direction of continuous casting. SEM analysis revealed that redistribution of Sn solute occurred, which was the reason for the solid grain nucleation near the walls of the two-phase zone mould. Luo et al. [37] have also proved that the liquid enriched Sn solute caused by solid grain growth will increase the amount of Sn solute at the solid–liquid interface.

Luo et al. [38] used Cu-4.7 wt%Sn to determine the flow of fluid and heat during the TZCC process. An alloy, 10 mm in diameter, was selected for this purpose. The speed of continuous casting, melting temperature, mould temperature, and the cooling water temperature were set to 20 mm/min, 1200 °C, 1040 °C, and 18 °C, respectively. The temperature of the alloy varied from 720 to 1081 °C. A complex circular flow caused by convection was observed in the mould. This facilitated the flow of the liquid alloy in the downward direction along the wall of the mould, followed by an upward flow in the centre. Analysing the direction of heat flow revealed that, at the centre, the flow was vertically downwards; at the upper wall, the flow was obliquely downwards, which was deflected towards the mould; and at the lower wall, the flow was deflected away from the mould.

### 9.3. Continuous Casting

The continuous casting of bronze alloys has an intrinsic advantage in terms of mechanical properties over other manufacturing processes because of the chilling and the sterling feeding of molten metal during solidification. Wilson [39] well-explained the continuous casting of copper-based alloys. Skoric et al. [40] provided numerical simulations and thermographic data on the structure of cast bronze alloy specimens regarding their stress status. Arsenovic et al. [41] also examined the effect of mould velocities on the fracturing of continuous cast specimens of bronze alloys [41]. Sergejevs et al. [42] suggested, a thorough description of the effect of casting velocity on the macrostructure and mechanical properties of Sn-bronzes. Due to the growing use of shape memory alloys (SMA), Kostov et al. [43] experimented on continuous cast copper-based shape memory wire with a diameter of 8 mm. Considerations of EN 1982:2008 [44] proved that macro segregation would be present in the resultant alloy after continuous casting. These macro segregations were responsible for the weakening of the alloy. Similar experiments carried out by Luo and He [45] examined the effect of process parameters on exudation thickness in continuous solidification of tin bronze. Another article by Luo [46] explained in further detail the influence of different continuous casting speeds on alloy microstructure evolution. They proved that, with a continuous casting speed increase, columnar crystals gradually replaced equiaxed crystals as the main component of the continuous unidirectional solidification of tin bronze. The diameter of the columnar crystals also begins to increase when the continuous casting speed is further increased. The effect of the distinct convection types, such as thermal buoyancy flow, solute buoyancy flow, inlet flow, and feeding flow on the formation of macrosegregation was examined by Ludwig et al. [47]. Increasing mush permeability will cause positive macrosegregation between the wall and centerline along with a negatively characterized macro segregation near the wall and in the centre. By contrast, when decreasing the permeability of the mushy zone, there will be positive macrosegregation at the wall along with a negative type of macrosegregation existing at the centreline. Grasser et al. [48] investigated the micro-macro segregation prediction based on solidification simulations for continuous casting of ternary bronze alloys. Moreover, Sugita et al. [49] utilized metal and sand moulds to experiment with solidification characteristics when casting processes were performed on Cu-20% Sn bronze alloys.

### 9.4. Semi-Continuous Casting and Die Casting

A significant factor that governs the material properties of bronze alloys is microstructure homogeneity. Therefore, Hao et al. [50] proposed a numerical analysis and an experimental investigation into solidification phenomenon during semi-continuous casting on phosphorous bronze alloys. To understand the solidification of these alloys, the Cu-Sn-P ternary method was investigated. Data from diffusion and annealing experiments were compared with Calphad-based computational thermodynamics. For the multiphase solidification simulation, thermodynamic information was used, considering the relative velocity and microsegregation. Feeding flow-induced macro-segregation was predicted as solvent accumulation at the surface and as depletion at the centre of the casting.

Backman et al. [51] explained the thermal behaviour of die casting for high-temperature alloys as being partly solidified during machine casting. Using machine die casting, partly solidified bronze alloy 905 was prepared. Using computer simulations for both liquid and partly solid alloys, die thermal behaviour was noted. Castings were either made in a laboratory system with low pressure or in a commercial die casting system with high pressure. Using a correlation between the measured die temperatures at the casting die interfaces and computer projections, the heat transfer coefficient was determined. Various advantages were demonstrated for the use of partially solid charge materials in a die casting machine at high temperatures. By casting the bronze alloy in a partly solid state, both the surface temperature and surface temperature of the alloy could be reduced. A sudden change in the coefficient of heat transfer was also observed when the die cavity was filled. By modifying the structure of the charged material (partly solidified) itself and recognizing its thermal behaviour to enhance the transfer of heat from the casting to the die, this study led to a new approach.

## 10. Property Variation in Different Compositions of Cu-Sn Alloy

### 10.1. Eutectic Composition of Cu-Sn Alloy

The creep behaviour of eutectic C-Sn alloy, with stress ranging from 10^−4^ to 10^−3^ at 303–393 K, was investigated in [52]. Initially, the presence of pure tin (β Sn) with a dendritic structure along with finely dispersed particles of Cu_6_Sn_5_ intermetallic compound (IMC) dispersed in pure tin was confirmed. The creep temperature changed from 0.55 to 1.00 Tm (melting temperature). This adjustment was made to limit the transformation of tetragonal pure tin into a cubic tin (tin) at 286 K. This study has also derived that the relation for apparent activation stress exponent (Na) was found to be dependent on temperature (directly proportional) and stress was applied. The value of the apparent activation stress exponent of the alloy was also compared with pure tin. It was observed that there was an increment in the value of apparent activation stress when Sn was alloyed with Cu. The results from creep data of Cu-Sn alloy confirmed the value of the true stress exponent for creep as 7 since the slope magnitude level was 7. The experiments also showed that the six orders of magnitude for steady-state creep rate were found to range from 10^−3^ to 10^−8^ s^−1^ when the stress was within the range of 10^−4^–10^−3^.

### 10.2. Hypereutectic Composition of Cu-Sn Alloy

Sn-2.0 wt%Cu and Sn-2.8 wt%Cu alloys were prepared using a transient directional solidification system. Spinelli et al. [53] conducted the microstructural evaluation of these hyper-eutectic alloys for a wide variety of experimental tip growth rates and tip cooling rates. Cu_6_Sn_5_ IMC evolved into morphologies that were M or H-shaped. There were rod-like particles in the microstructure of the Sn-rich β matrix of both alloys (Sn-2.0 wt% Cu and Sn-2.8 wt% Cu). With minimum/maximum values of 16.0/37.0 mm and 2.0/12.5 mm for the respective alloys, inter-branch spacing differed significantly. For the alloy containing 2.0 wt% Cu, and 0.8 to 34.0 K/s for the alloy containing 2.8 wt% Cu, experimental cooling rates ranged from 2.7 to 33.0 K/s. In this research, Hall–Petch style equations that connected hardness, UTS, and elongation to fracture with inter-branch spacing were proposed. Similar patterns for hardness and UTS were illustrated by two alloys. For these two alloys, the ductility behaviour was found to be entirely different. The tensile strength analysis from this study has confirmed that after the process of transient directional solidification, the Sn-2.8 wt%. Cu alloy successfully meets the required mechanical strength.

### 10.3. Peritectic Composition of Cu-Sn Alloy

Peritectic transformations are demonstrated by several binary alloys during solidification. In these cases, the primary solid alpha phase precipitated from the liquid phase interacts at peritectic temperature with the liquid phase to form a peritectic beta phase. A few microstructures can be formed by binary alloys with a peritectic composition. Kohler et al. [54] investigated the microstructure of bronze alloys during low-speed peritectic solidification. Thereafter, Zhai et al. [55] presented different mechanical and microstructural properties of dynamically solidified peritectic Cu-70 wt%Sn alloy. These alloys were subjected to an external ultrasonic field with a frequency of 20 kHz and a 440 W power supply. The external ultrasound that is being induced promoted and completed peritectic transformation (L + ϵ→η). Mechanical properties, such as micro-hardness and compressive strength, were increased to a factor of 1.45 and 4.8, respectively. This was attributed to the increase in volume fraction and grain refinement. According to Zhai et al. [55], there were two methods to improve the microstructure resulting from peritectic compositions of Cu-Sn alloys. The first method was by increasing the rate of undercooling so that significant improvements in the primary phase could be achieved. If sufficiently high, undercooling stimulates the peritectic phase to directly nucleate from the metastable liquid alloy. The other method was to apply strong external fields, such as ultrasonic fields, to modify the peritectic solidification process.

**Figure 20:**
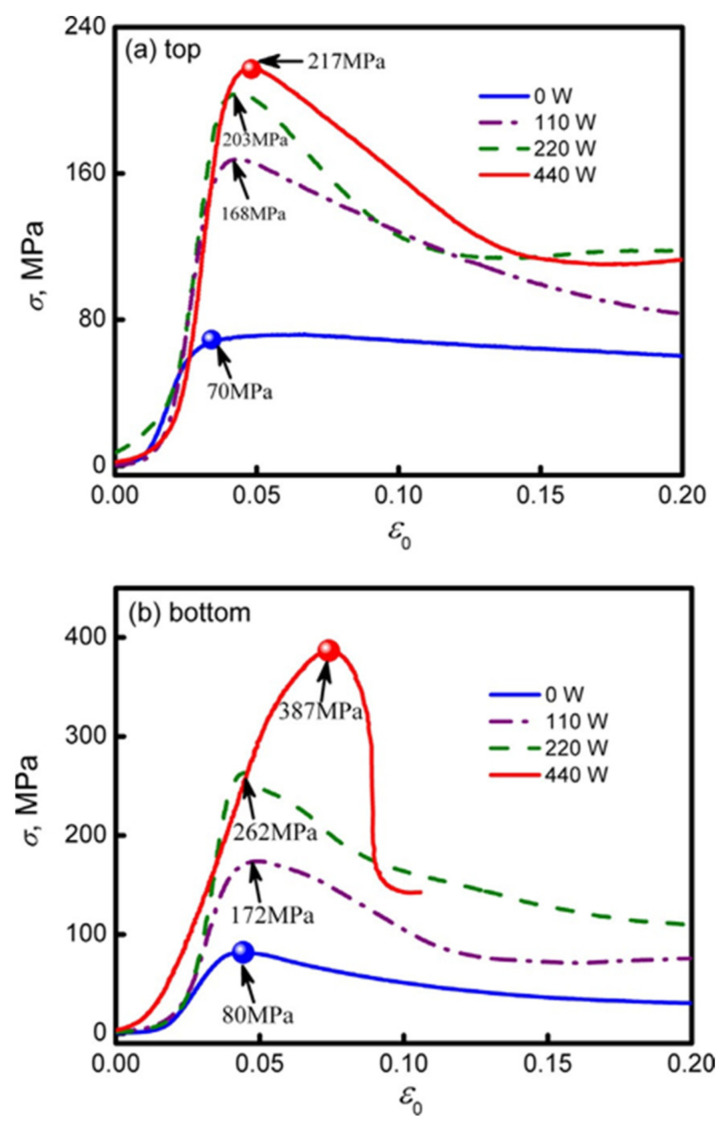
Compressive stress–strain curve for Cu-70 wt%Sn alloy that was solidified under static and ultrasonic conditions for the (**a**) sample top and (**b**) sample bottom [55].

The primary phase (ϵ) in the Cu-70 wt%Sn alloy was found to be Cu_3_Sn IMC. The peritectic product was also an intermetallic compound Cu_6_Sn_5_ (η). It was found that ultrasonic waves affected peritectic transformation in two ways. Firstly, by promoting the intercooling of the primary phase (ϵ) and preventing the excess undercooling of the liquid alloy. It was found that the grain size of the primary phase had been refined and turned into an equiaxed shape with enhancements when exposed to ultrasound power. Secondly, refinement of peritectic grain structure resulted in a large increase in the Fourier number, which stimulates the completion of the peritectic state transformation. Looking into this, Zhai et al. [55] proved that an ultrasonic wave can be used to enhance the mechanical properties of the peritectic grain. The stress–strain curves were plotted for the top sample and bottom sample (as shown in Figure 20). This was done because there was a difference in the compressive strength for each sample, caused by the attenuation of sound waves. Figure 20 confirmed that, with an increase in the power of the ultrasound, a significant rise in stress value was observed.

On Cu-14.5 wt% Sn, Cu-21.3 wt% Sn and Cu-26.8 wt% Sn peritectic alloys, Kohler et al. [56] conducted single-path analysis. To measure the changes in the solid mass fraction, latent heat and composition during alloy solidification, a new heat flow model coupled to a Cu-Sn thermodynamic database has been developed. With the microsegregation model, including back-diffusion in the primary solid phase, a close comparison was made. In hypoperitectic Cu-Sn alloys solidified at low speed in a diffusive regime, Valloton et al. [57] performed experiments to ensure planar growth of the primary and peritectic phases. To minimize convection, samples with a reduced diameter equal to 500 µm were taken. The laminar and fibrous cooperative growth of the primary alpha and peritectic β-phases with a length of several millimetres was also found to be formed. Within a high peritectic alloy solidification interval, a quenched alpha + β front was achieved. These morphologies were observed under SEM for peritectic systems with a temperature of just a few Kelvins in the freezing range. The primary phase’s high-volume fraction, differing over time, was found to be close to the quenched surface. This shows that the cooperative production front never entered a steady state. In addition, a link was formed between the primary FCC phase and the peritectic bcc phase. This was achieved to promote the peritectic process with multiple primary phase enucleations.

## 11. Applications of Cast Bronzes

**Figure 21:**
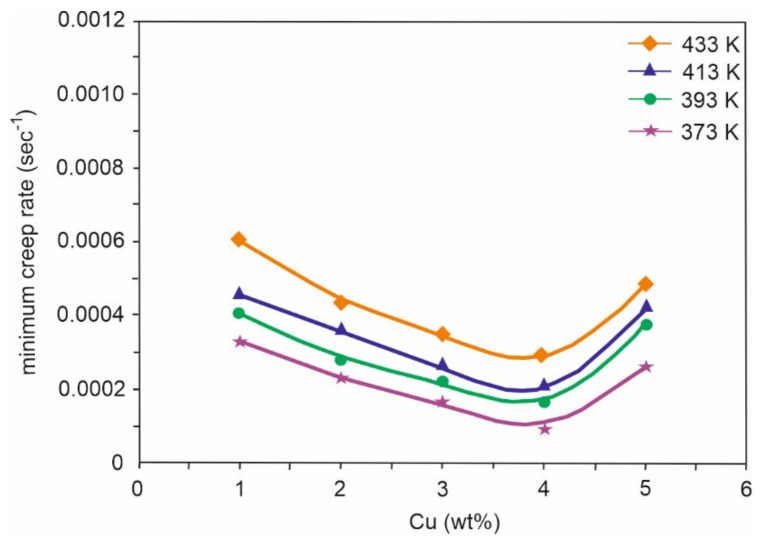
Variation of creep rates concerning change in wt% of Cu [59].

**Figure 22:**
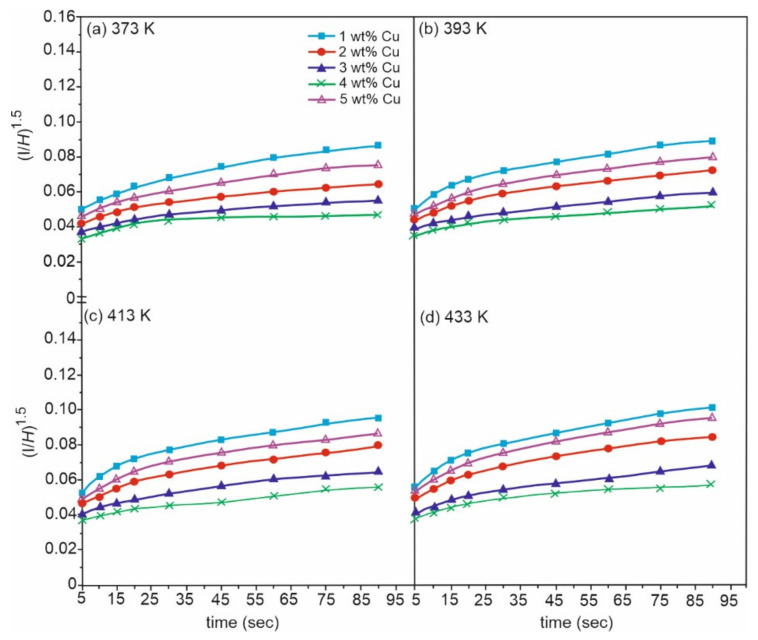
Variation of creep rate at increasing annealing temperature [59].

**Figure 23:**
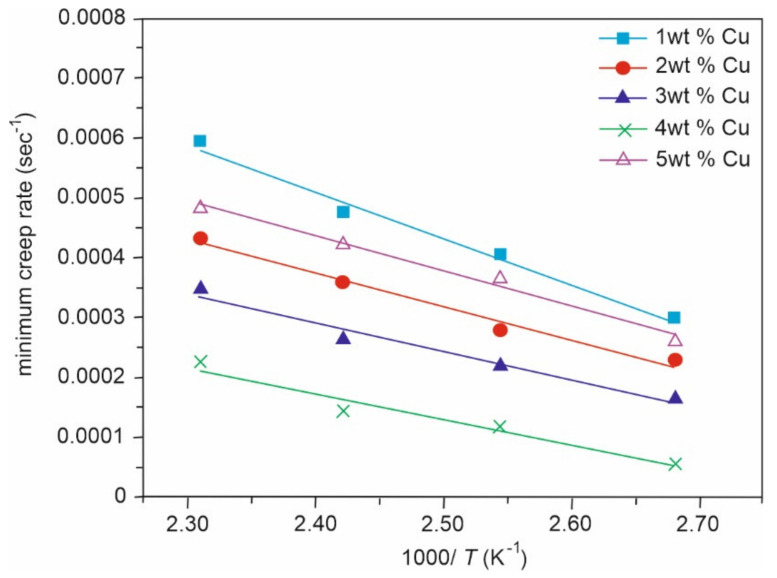
Variation of minimum creep rates with 1000/T, where T is the respective annealing temperature [59].

**Figure 24:**
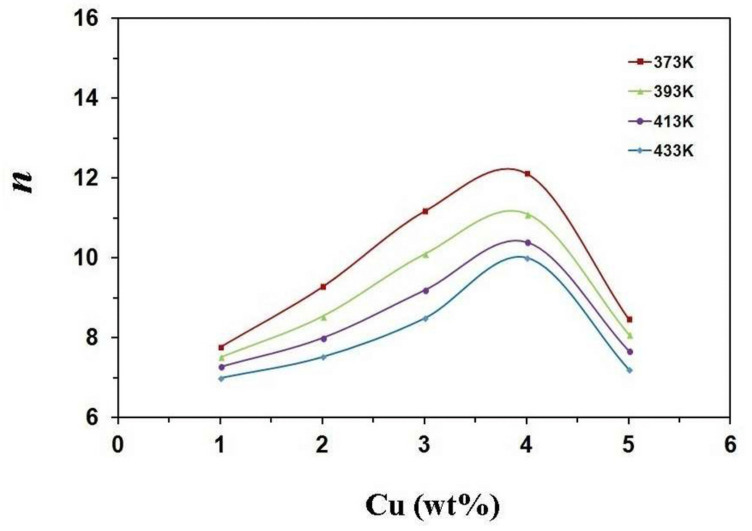
Plot between stress component (n) and wt% of Cu [59].

Sn-Cu alloys are a potential substitute for soldering materials due to Pb toxicity, thanks to their good wettability, high electrical conductivity, good mechanical properties, and low cost. Electronic interconnections can be established by incorporating bronze alloys. Gain et al. [58] investigated the morphology and impact of Cu_6_Sn_5_ intermetallic compound on Sn-0.7Cu alloy properties at high temperatures. In the as-cast condition, there was the presence of fine Sn grains. Furthermore, hardness, electrical property, and creep behaviour were improved due to the obstruction of dislocation motion caused by the intermetallic presence at the grain boundaries (0.2–0.3 µm in diameter). After heating, the intermetallic compound grew in size (of about 915 µm) and was found to be coarsened and elongated. Finally, due to the coarsening of intermetallic compounds, Gain et al. [58] concluded that the hardness and electrical property of bronze were degraded to 18.5% and 12%, respectively, along with an increment in damping property. Hence CuSn alloys are an efficient replacement for lead alloys, that are mainly used for soldering purposes. Detailed experiments were conducted and an experiment was carried out by Rehim et al. [59]. Their research covered the microstructure and mechanical properties of five variants of bronze alloys, Sn-x Cu, where the content of Cu varied from 1–5 wt%. Heat treatment at 373, 393, 413, and 433 K for 2 h proved an increase in minimum creep rates, whenever the wt% of Cu was increased to 4 wt% (as shown in Figure 21) or on increasing the ageing temperature (as shown in Figure 22). After incorporating SEM and XRD techniques, a plot for minimum creep rates was also carried out as shown in Figure 23. These plots confirmed that Sn-4 wt% Cu alloy exhibited the best creep resistance. Furthermore, the variation in the stress component with increasing Cu content was also studied and plotted, as shown in Figure 24. This plot confirmed that stress was limited in the component whenever the annealing temperature was forced to increase. Here, a component with minimum stress resulted when the annealing temperature was set at 433 K.

The corrosion activity of intermetallic Cu-Sn was examined at 3.5 wt%. by using galvanic corrosion and polarization processes, percent of NaCl solution, and was compared with Cu and Sn. Even though stainless steel is of superior quality, the study conducted by Tsao et al. [60] verified using of bronze alloys in the development of underwater bearings and ship propellers. The Sn, Cu, Cu_3_Sn, and Cu_6_Sn_5_ polarization curves clearly showed that an increase in Cu material correlated with a substantial increase in corrosion current density. In the NaCl solution, corrosion values and the corrosion phase of Cu-Sn showed that Cu species diffusion occurred through the oxide layer. It was found that Sn_3_O(OH)_2_Cl_2_ and CuCl, respectively, were the corrosion products occurring on the surfaces of pure Sn and Cu. In addition, the following corrosion products coexisted, Cu_3_Sn, Sn_3_O(OH)_2_Cl_2_ and CuCl. The formation of various compounds, including SnO_2_, Sn_3_O(OH)_2_Cl_2_, Cu_2_O, and CuCl_23_Cu(OH)_2_ was found due to the presence of the corrosion compound Cu_6_Sn_5_. Once the polarization tests were completed, there was an absence of SnO and CuCl compounds.

Liu et al. presented research [61] covering the microstructure and mechanical properties of Cu-Sn alloys for the development of detonating and primer cords. Due to its non-toxicity and abundance of resources, Cu-Sn alloys have been chosen for this reason. Alloys based on Cu-Sn have a high density, which was useful for supplying penetration momentum and impulse energy. Sn (0.3–1.0) wt% Cu alloy was chosen for the experiment. The phase constituent of the alloy was not altered by rolling, whereas the microstructure was significantly refined. It was observed that annealing does not affect the rolled Cu-Sn alloys. However, this increased the ductility and decreased the strength of the annealed alloy. On testing rolled Cu-Sn alloy, acceptable tensile properties for the purpose were attained. This exhibited strain-softening, which is desirable for the processing of cords.

Cu alloys with solid lubricant (mainly Pb-bronze) can be efficiently employed in anti-seizure applications. Sato et al. [62] have investigated tribological behaviour and the properties of sulphide-dispersed bronze. Friction testing under dry conditions yielded improved results for properties such as hardness and wear resistance. Sulphide-bronze prevents seizures only in certain manually set conditions, whereas sulphide was the reason for the suppression of scoring and seizure. Limited or restricted movement of Cu alloy components to the matrix steel surface by dispersed sulphide can also be attributed to the increase in hardness and wear resistance. Furthermore, adding Si to the Cu-Fe-S system and sulphide-bronze results in crystallizing the Fe-Si compound.

## 12. Evolution of Cast Cu-Sn-Ti Alloys

Sn content in the bronze alloy can vary from 2 wt% to 20 wt%. In Cu-Sn alloys, the amount of tin plays a major role, affecting its strength and usability. The high price of tin increases the cost of the final product. For this reason, to achieve desired properties and to be cost-effective, other allowable additives were introduced, such as zinc, lead, phosphorus, nickel, iron, titanium, aluminium, etc. Adding aluminium to bronze results in increased strength and hardness and a decrease in the level of plasticity. Research on Cu-Sn alloys with aluminium was conducted to optimize the amount of aluminium addition. Furthermore, the addition of iron in Cu-Sn alloys changes the properties of the alloy. Hence, a study based on the above-mentioned topic was put forward by Klempka et al. [63]. Both alloying elements, aluminium and iron caused grain refinement in Cu-Sn10 alloys. The major change observed due to the introduction of 0.8 wt% Al and Fe was the rise in UTS and hardness. A rise in UTS was higher when iron was added as compared to the addition of aluminium. The addition of both these alloying elements decreases the alloy plasticity. Considering plasticity, the drop due to aluminium addition was more significant with the addition of iron.

Titanium was introduced into bronze alloys because of its excellent stability and tensile properties. Therefore, the effect of Ti on bronze alloys was thoroughly investigated. An increase or decrease in reaction rate can be obtained in between solder and joining metal by adding elements to the interface layer. Moreover, this addition can alter the physical properties of phases present at the interface and can lead to the formation of new phases or new layers. Vuorinen et al. [64] performed solid-state annealing of Cu-Sn alloys and added certain amounts of Ti to investigate the behaviour of Ti on parent metal with annealing periods of up to 3000 h. Initially, the addition of Ti does not correlate with the thickness of the intermetallic compounds (Cu_6_Sn_5_ and Cu_3_Sn) formed at the interface. Hence, the thickness of those intermetallic compounds was unaffected by the addition of Ti, even though an unevenness was developed. As per the EDS resolution limits, there was no observance of Ti in both of the intermetallic compounds. Further additions of Ti cause the heterogeneous distribution of both layers. Due to the non-reactive nature of Ti to intermetallic (Cu-Sn), this forced them to react with Sn to produce large platelets of Ti_2_Sn_3_ observed in the solder matrix. Hence, any addition of Ti to the matrix does not affect the Cu-Sn intermetallic formed at the interface. Rationalizing this behaviour with the phase diagram ascertains that Ti possesses very low solubility for Cu-Sn IMCs.

The results of solidifying binary Cu-Sn and ternary Cu-Sn-Ti alloys at a lower cooling rate in a differential scanning furnace were compared to fast solidification of these alloys in the experiment conducted by Li et al. [65], and properties, such as alloy composition and cooling rate, were studied. Considering binary alloys, a metastable Cu_5.6_Sn phase was observed at a high cooling rate, which was obtained from the parent β phase by diffusing less martensitic transformation. Ti was observed to form (CuSn)_3_Ti_5_ in ternary alloys in low and elevated cooling rate conditions. By having the binary system in rapid solidification conditions, the microstructure of Cu-Sn was composed of Cu5.6Sn and primary α dendrites. Considering the ternary system, (CuSn_3_)Ti_5_ was formed without decomposing into other ternary compounds. This was attributed to rapid cooling, which resulted in the blockage of solute diffusion in solids. When increasing the Ti content, the amount of (CuSn_3_)Ti_5_ and α phase were increased, thereby decreasing the content of Cu_5.6_Sn.

## 13. Properties of Cu-Sn-Ti Alloys

**Figure 25:**
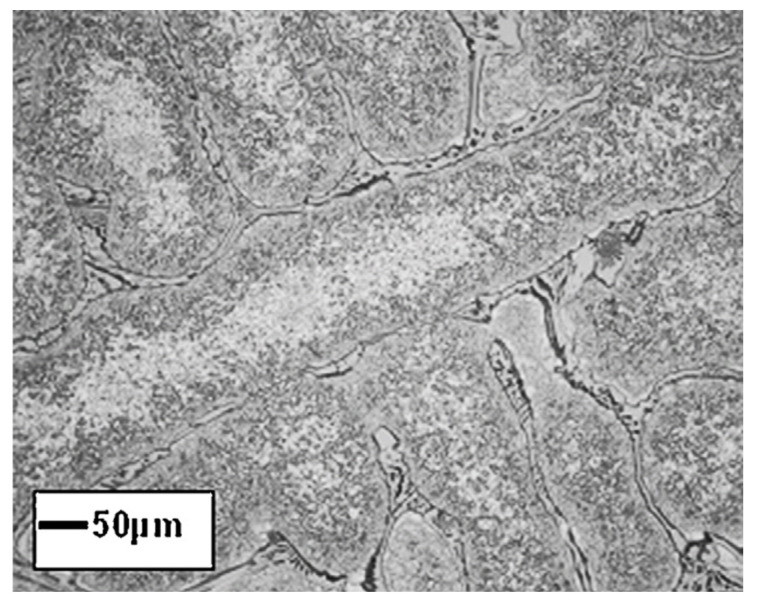
Microstructure of CuTi_3_Sn_2.75_ alloy under OM in as-cast condition [67].

**Figure 26:**
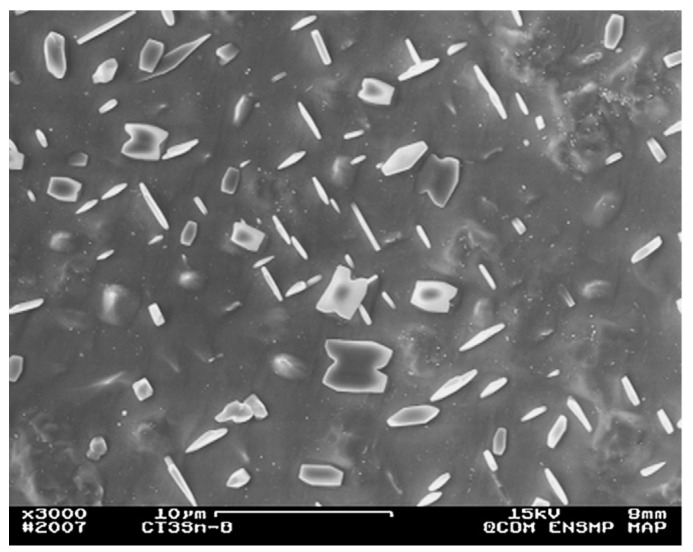
Observation under SEM, proving the presence of fine precipitates in the core of dendrites [67].

The wettability of a solid substrate by a liquid metallic material is crucial to understanding the bonding characteristics and the interactive forces at the interface. To ensure good wetting characteristics, fluxes are used. The development of new fluxes requires studying surface tension. The wetting characteristics and mechanical properties of brazing metals, such as Cu-Sn, can be enhanced by adding a small amount of titanium. The surface and transport properties of Cu-Sn-Ti liquid alloys were studied by Novakovic et al. [66]. It was found that the addition of titanium greatly improved the wetting characteristics and mechanical properties of Cu-Sn binary alloys. Using quasi-chemical approximation and compound formation methods for binary systems, concentration and temperature dependencies of surface tension and surface composition were analysed. In the binary systems, Cu and Sn-atoms segregate in bulk compositions. All binary systems tended to form a compound. The thermodynamic property of all three binary systems exhibited a negative deviation from Raoult’s law, while the surface tension isotherms exhibited a positive deviation. For the ternary system, only the surface tension has been found and the result was compared with corresponding Cu-Sn surface tension data. Moreover, alloys that were rich in copper were investigated by Lebreton et al. [67], to analyse their microstructural and mechanical properties. Three alloys, CuTi-2 wt%Sn-2.75 wt%, CuTi-3 wt%Sn-2.75 wt% and CuTi-4 wt%Sn-2.75 wt%, were selected for this purpose. CuTi_3_Sn_5_ intermetallic eutectic ternary compound was confirmed, as well as the product characteristics during the as-cast conditions. Furthermore, this compound could not be further dissolved into the matrix phase using any thermomechanical processes. A Ti content of more than 3 wt% was needed to obtain the required amount of hardness due to the decreased spinodal decomposition. Figure 25 and Figure 26 prove that the microstructure of the dendritic structure was characterized by the formation of fine precipitates. All three samples had the same microstructure, with fine spherical precipitates that evolved from a rod-like form, with limited discontinuity in the resultant alloy. Small additions of Cr into CuTi_4_Sn_2.75_ resulted in enhancing hardness for the first 2 h along with the elevation of phase transformation temperature of the alloy. Through XRD patterns of CuTi-3 wt%Sn-2.75 wt%, the existence of a metastable phase limiting the coalescence was confirmed. This was the main reason for the softening of alloys rich in copper.

The experiments conducted by Lin et al. [68] showed that the minimum amount of Ti to efficiently wet alumina was found to be 6 wt%. When increasing the amount of Ti from 6 to 12 wt%, there seemed to be an increase in the volume fraction of the intermetallic phase and wettability enhancing the bond strength. Results from sessile drop tests confirmed that 70Cu-21Sn-9Ti was the best compound composition to wet alumina. Studies conducted by Lin et al. [68] provided a note to minimize the amount of Sn to below 21 wt%. At 900 °C, the best wettability was obtained when Ti content was set to 9 wt%, whereas increasing content to 12 wt% did not seem to enhance the wetting behaviour. Moreover, thermal expansion coefficients of Cu-Sn-Ti alloys were compared with Ticusil^®^ alloy and were found to be lower. It was also observed that a combination of a lower coefficient of thermal expansion and lower wetting angle could be obtained using Cu-Sn-Ti alloy, which confirmed its dominance over commercially used Ticusil^®^ alloy. Therefore, a minimal amount of residual stress can be expected when brazing alumina with Cu/Sn/Ti alloys.

## 14. Phase Diagrams and Phase Relations of Cu-Sn-Ti Ternary System

**Figure 27:**
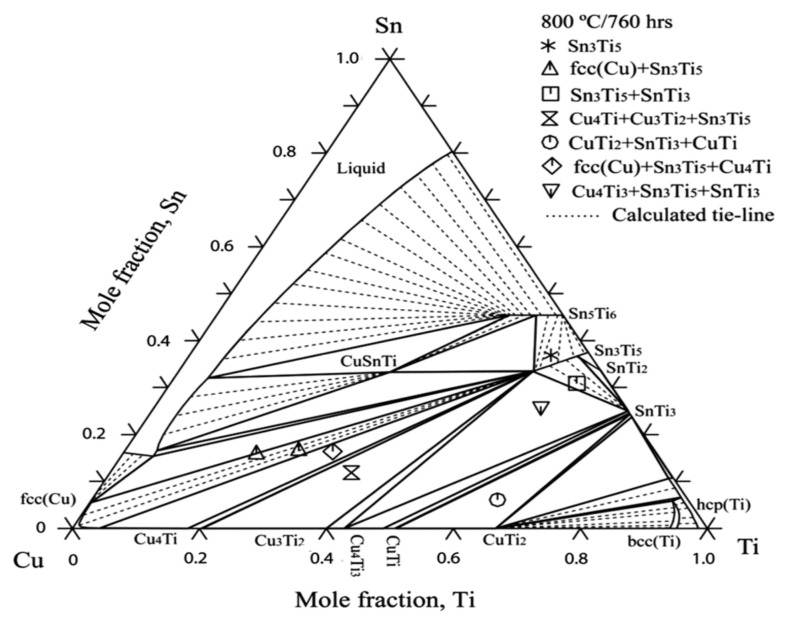
Ternary system of Cu-Sn-Ti ternary system at 800 °C [69].

**Figure 28:**
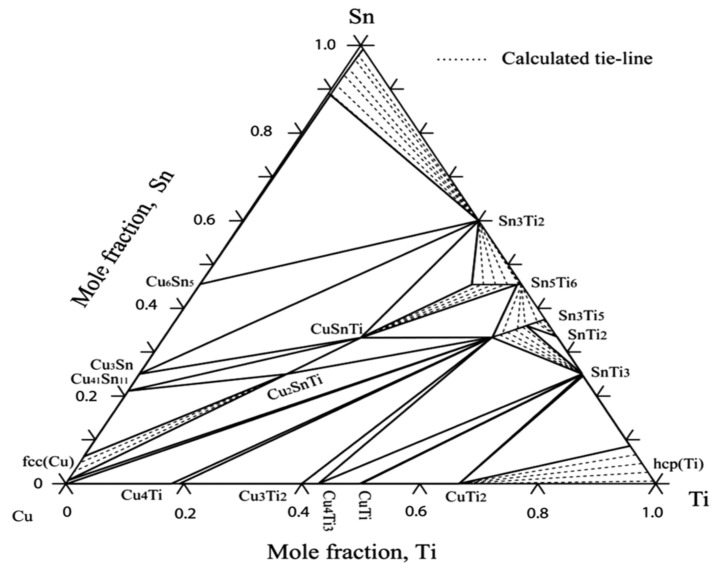
Isothermal section of Cu-Sn-Ti ternary system at 697 °C [69].

**Figure 29:**
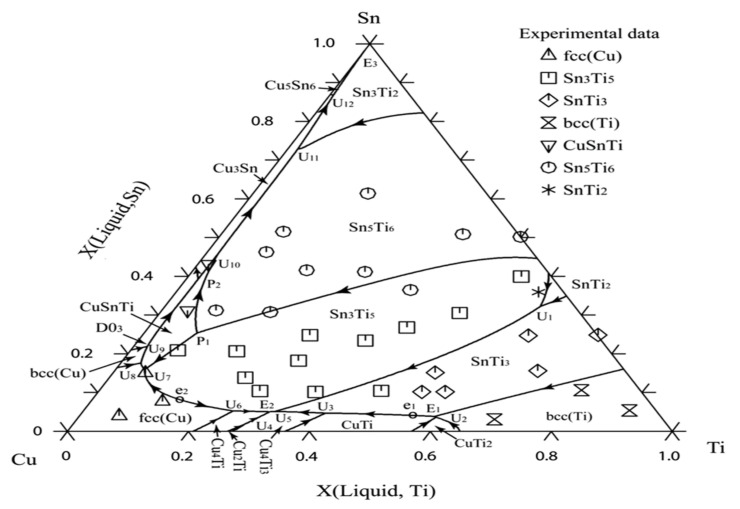
Liquidus projection of Cu-Sn-Ti ternary system superimposed with experimental data of primary solidification [69].

To understand the complex interface reaction during melting and casting and to get adequate mechanical properties, the knowledge of the thermodynamic properties and phase equilibria of Cu-Sn-Ti is essential. Wang et al. [69] obtained experimental data for Cu-Sn-Ti phase equilibria at 800 °C. Wang et al. [69] concluded that one single-phase point, three tie lines and four tie triangles were present, as shown in Figure 27, by integrating Euclidean distance matrix analysis (EDMA). Hence, the solid phase equilibrium calculations at 800 °C affirmed the presence of a binary compound (Sn_3_Ti_5_) which possess an extended solubility of Cu. Another objective of this study was to define binary systems present at 397 °C, as depicted in Figure 28, in the isothermal section and to obtain a collection of accurate and self-consistent thermodynamic parameters for this ternary Cu-Sn-Ti system. The solubility ranges of Sn-Ti alloys (Sn_3_Ti_5_ and Sn_5_Ti_6_ compounds) and the presence of two ternary compounds (CuSnTi and Cu_2_SnTi) have been verified, along with the evaluation of liquid and FCC phase interaction parameters. The findings were extremely accurate on the Cu-rich side of the ternary method and can be used effectively to make decisions on Cu-based alloys high temperature (brazing) applications. The determined liquid projection of the Cu-Sn-Ti ternary system superimposed on the experimental primary phases is shown in Figure 29. Solubility ranges of the two major binary compounds have also been depicted in the figure.

**Figure 30:**
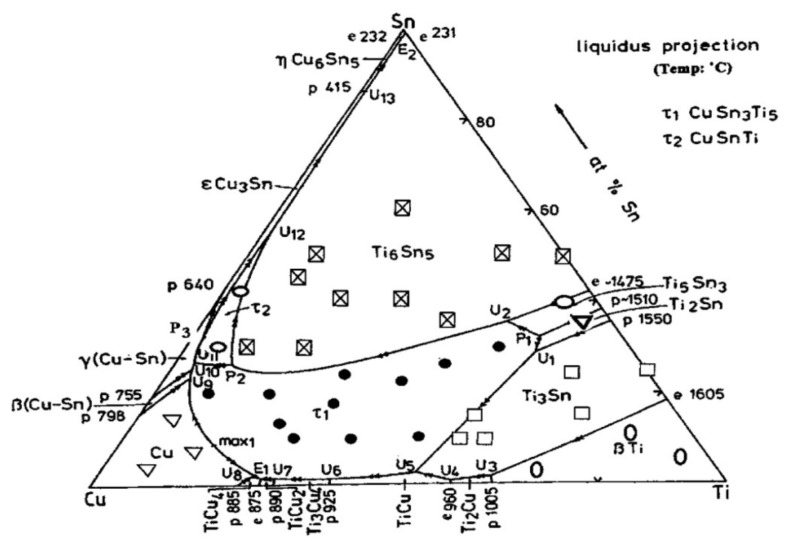
Liquidus surface projection in Cu-Sn-Ti ternary system [70].

The formation of the CuSn_3_Ti% intermetallic phase is the main reason for the degradation of strength whenever CuSnTi filler metals are used. To predict the brazeability of CuSnTi filler metals, there is an increased need to analyse the liquidus of the CuSnTi ternary system. Naka et al. [70] came up with an experiment to sketch the liquidus of the CuSnTi ternary system. Their results lead us to the conclusion that, as shown in Figure 30, the Cu-Sn-Ti alloy method yielded a large region of primary crystallization of phases that were Cu, Ti_6_Sn_5_, CuSn_3_Ti_5_, and Cu-Sn-Ti. Due to the peritectic response, both ternary compounds were formed. Near the Sn-rich portion of the phase diagram, a ternary eutectic L = Sn + ƞ (CuSn) + Ti_6_Sn_5_ was observed. Moreover, a pseudo-binary eutectic reaction accompanied the formation of Cu+CuSn_3_Ti_5_.

**Figure 31:**
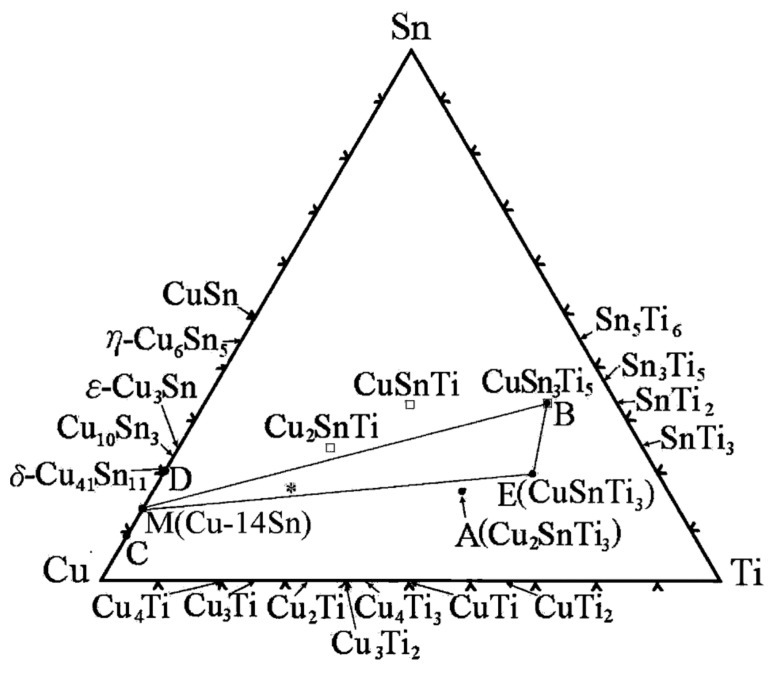
The phase relationship of constituent phases of Cu-23at. %Ti-17at. %Sn alloy in the ternary phase diagram of Cu-Sn-Ti when isothermally held for 10 h at 900 °C [71].

Huang et al. [71] isothermally treated Cu-23at. %Ti-17 at. %Sn at 900 °C for 10 h and then quenched it in water. This study has revealed interesting facts about the intermetallic compounds formed after the commencement of the reaction. This resulted in a new ternary compound of Cu, Sn and Ti, forming CuSnTi_3_. Figure 31 depicts the phase relationship of Cu-23at. %Ti-17at. %Sn in the Cu-Sn-Ti ternary system. Overall, alloy constituents observed after 100% commencement of reaction included Cu-14at. %Sn, CuSnTi_3_, CuSn_3_Ti_5_ and Cu_2_SnTi_3_. When cooling the furnace, Cu-14at. %Sn decomposed into Cu-9 wt%Sn and Cu_41_Sn_11_. Similarly, Cu_2_SnTi_3_ tended to decompose into Cu and Cu-Sn-Ti when subjected to prolonged heat treatment. The only thermodynamically stable compound present at 900 °C was CuSnTi_3_ with a hexagonal structure (a = 0.4636 nm, c = 0.5229 nm). Furthermore, it was found to be iso-structural with Ni_3_Sn_2_ having Cu and Sn distributed in the lattice positions of 2c (at x = 1/3; y = 2/3; z = 1/4; occ = 0.5) and Ti being distributed in 2a (at x = 0; y = 0; z = 0; occ = 1) and 2d lattice positions (at x = 1/3; y = 2/3; z = 3/4; occ = 0.5).

## 15. Effect of Elemental Additions on Cu-Sn-Ti Alloys

Cu-Sn-Ti alloy can be effectively employed to make abrasive binders. The electron microscope examination provided the image of the columnar structure of Sn and Ti near the abrasive materials (diamond or CBN grains) and the coarse particles of metal, thereby owing to an irregular microstructure of the resulting alloy. This will adversely affect the strength and ductility of the compound. The variation of properties on adding refractory metals (V, Mo, B, and Re), refractory compounds (LaB_6_, TiC, TiN, Si_3_Ni_4_), and rare earth metals (La, Y) to the ternary system was investigated by Kizikov et al. [72]. Mechanical properties were improved when 0.01% of Si_3_N_4_ was added to the matrix. The micro additions of 0.01% of Si_3_N_4_ to Cu-20Sn-10Ti led to a structural modification, which further contributed to higher strengthening behaviour of the resultant alloy. Moreover, it was also confirmed that the micro additions of 0.01% of compounds improved strength and ductility. Tsao et al. [73] prepared four samples of Ti_7_Cu_x_Sn (where x = 0, 1, 2.5, 5) alloy, solution treated (ST) them for 2 h at 1000 °C and quenched them in water at room temperature. Results confirmed the presence of a martensitic structure in ST Ti_7_Cu alloy. Further Sn additions to Ti_7_Cu_x_Sn refined the microstructure of Ti_7_Cu_x_Sn alloy and resulted in a pseudo dendritic microstructure consisting of α Ti-phase. Potentiodynamic polarization curves and electrochemical impedance spectroscopy results also showed that Sn additions greatly improved the corrosion-resistant behaviour of the alloy because of the formation of a dual-layer oxide with an internal barrier and a porous external layered surface. Thus, it can be inferred that additions of Sn into ST Ti_7_Cu_x_Sn alloys will result in an improvement of electrochemical corrosion resistance.

**Figure 32:**
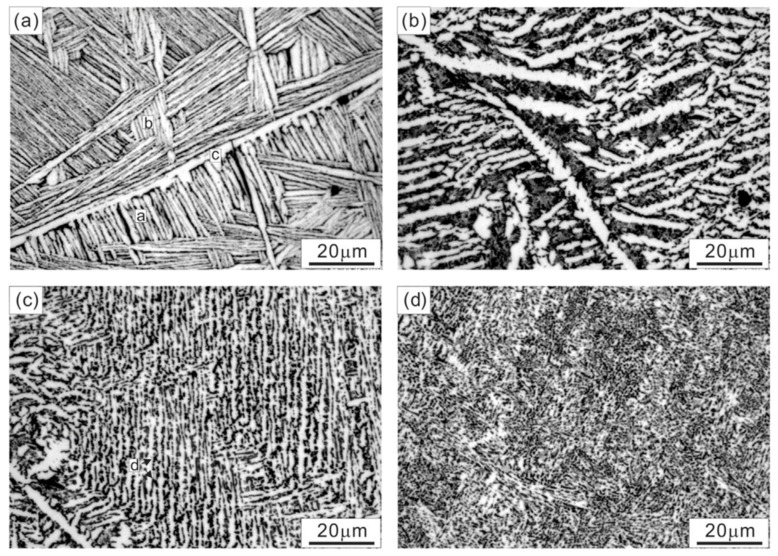
Microstructure of (**a**) Ti_7_Cu, (**b**) Ti_7_CuSn, (**c**) Ti_7_Cu_2.5_Sn, (**d**) Ti_7_Cu_5_Sn under OM [74].

**Figure 33:**
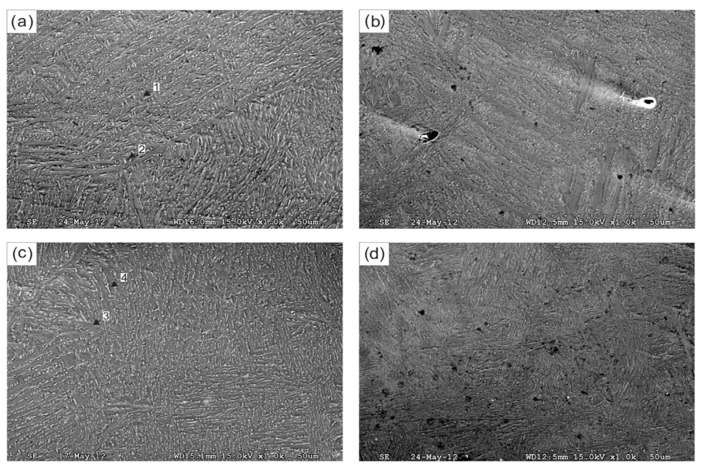
Microstructures under SEM after potentiodynamic testing in (**a**) Ti_7_Cu, (**b**) Ti_7_CuSn, (**c**) Ti_7_Cu_2.5_Sn, and (**d**) Ti_7_Cu_5_Sn alloy [74].

Another experiment on the effect of Sn content on the microstructure and corrosion behaviour of Ti_7_Cu_x_Sn (x ranges from 0–5 wt%) was put forward by Tsao [74]. A corrosion test was conducted with 0.9 wt% NaCl solution at 25 °C. Potentiodynamic polarization curves and electrochemical impedance spectroscopy (EIS) results confirmed that the addition of up to 5% Sn improved the corrosion resistance of Ti_7_Cu_x_Sn alloy, which also exhibited a dual-layer consisting of the porous outer covering and an inner barrier layer with oxide content. The microstructure before and after potentiodynamic testing was also obtained by the incorporation of OM (Figure 32) and SEM (Figure 33), respectively. Therefore, an increase in the volume fraction of the Ti_2_Cu stage was observed by eliminating a basket-wear type of structure, thereby promoting an ultra-fine structure consisting of α-Ti and Ti_2_Cu in the Ti_7_Cu_5_Sn alloy.

**Figure 34:**
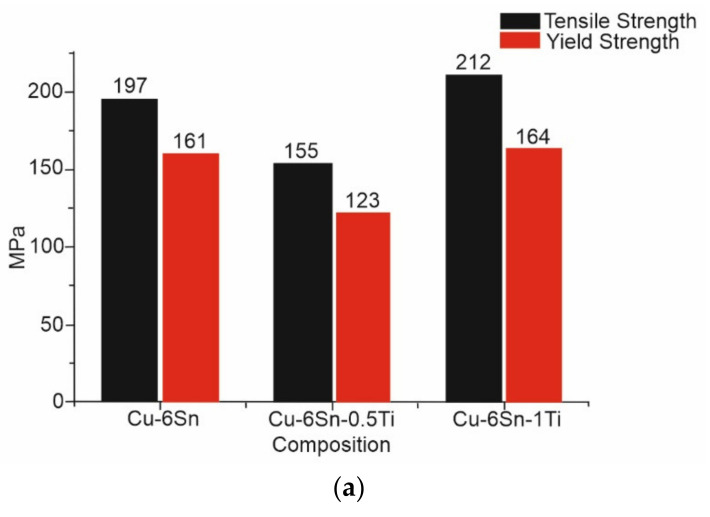

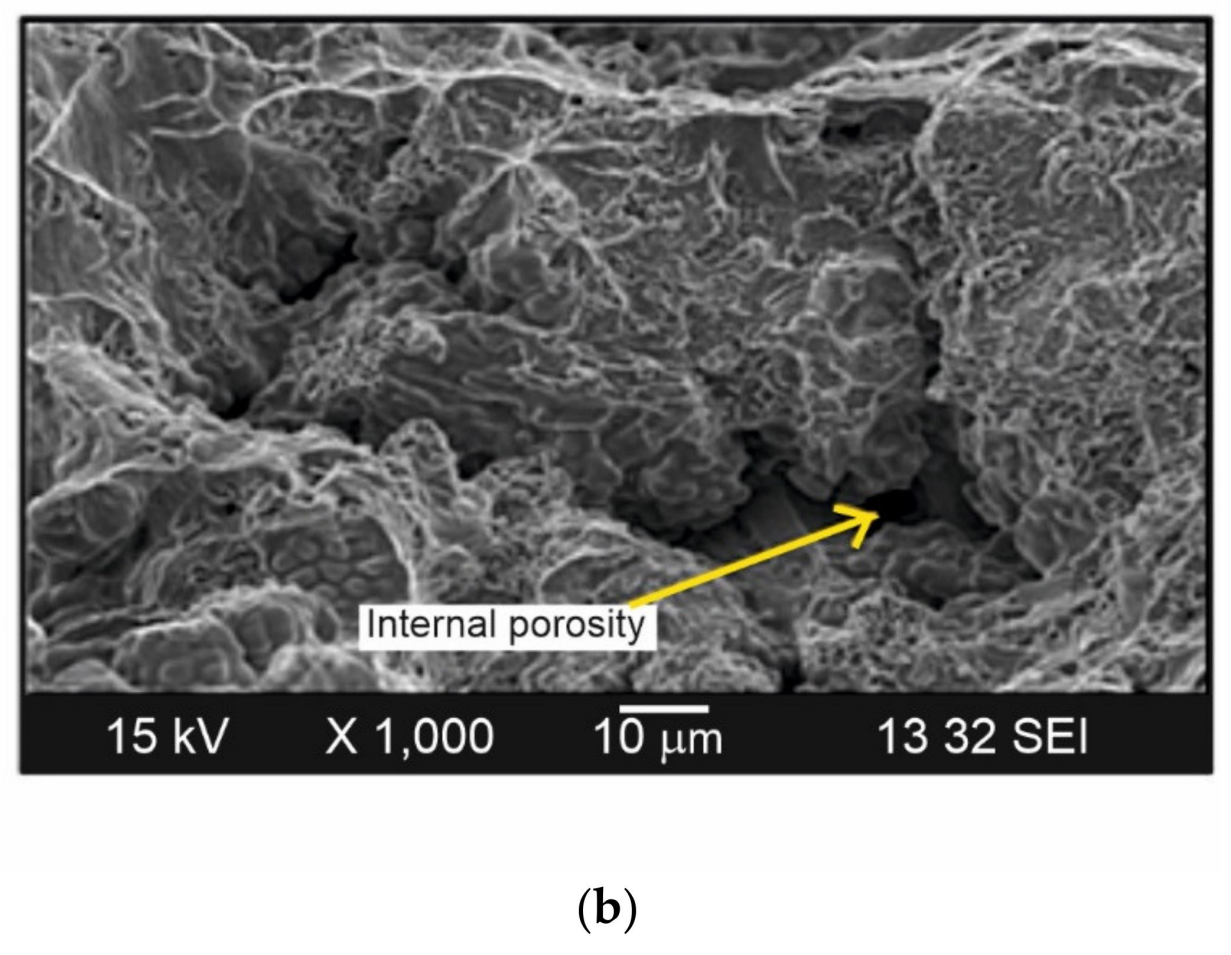
(**a**) Comparison of yield strength and tensile strength of as-cast alloys, (**b**) SEM image of a fracture surface of Cu-6Sn-0.5Ti [75].

The effect of Ti additions on as-cast bronze alloys was investigated by Akshay et al. [75,76]. The impact of adding small amounts of Ti on the microstructure, mechanical properties and tribological properties was recorded and compared with that of as-cast Cu-6Sn. Due to the development of blowholes, the tensile properties were found to reduce by up to 0.5 wt%. of Ti additions. Figure 34b depicts the presence of internal pores at the fracture surface of Cu-6Sn-0.5Ti alloy. Furthermore, on increasing the content of Ti addition to 1 wt%, the formation of the intermetallic compound was observed in Cu-Sn-Ti alloy, which was attributed to its high tensile strength and improved hardness (dendritic structure with fine grains). A comparison of the tensile strength and yield strength for three different as-cast Cu-Sn-Ti alloy compositions are presented in Figure 34a. By using a pin-on-disc tribometer with load variations varying from 10 to 30 N, a sliding distance of 1000 m, and sliding velocity of 1–3 m/s, tribological investigations showed that there was a rise in wear rate with increasing load or decreasing sliding distance. Hence, the wear resistance increased with Ti additions in the bronze alloy. Moreover, the mode of failure shifted from ductile to brittle as dimples were visible under microscopic observations with additions of Ti. This also confirms that Cu-Sn-Ti alloy can efficiently replace bronze alloy when working conditions are intense and high resistance to wear is required.

## 16. Cu-Sn-Ti Brazing Alloys

Cu-Sn-Ti based brazing alloys are widely used for joining materials, such as diamond/steel matrix tools [77,78,79,80,81,82,83], metal–ceramics [84], and CBN abrasive tools [85,86,87]. The most common alloys previously used for brazing were alloys based on Ag-Cu-Ti. However, as pointed out by He et al. [88], these alloys had a low strength, high cost, and low wear resistance. Cu-Sn-Ti alloys were observed to have excellent mechanical properties, comparable high strength and a low cost, thus making them suitable for active braze alloys, according to many researchers [68,83,84].

**Figure 35:**
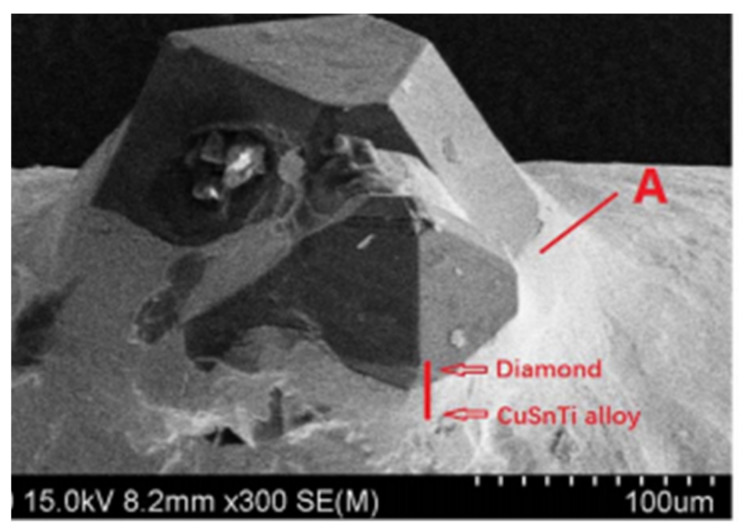
Interface microstructure between Cu-Sn-Ti filler alloy and diamond grits [79].

**Figure 36:**
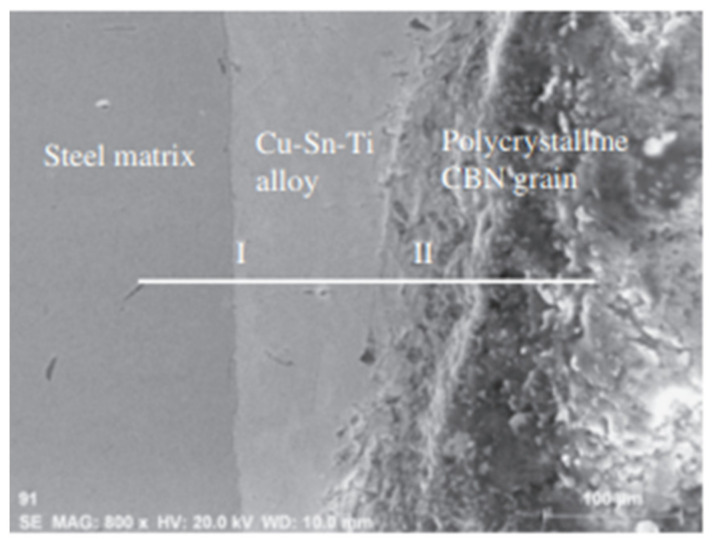
Interface microstructure of steel matrix/Cu–Sn–Ti alloy/polycrystalline CBN grain [84].

Presently these alloys have been gaining importance as active braze alloys, especially in diamond/metal and polycrystalline CBN/metal tools. Studies have observed the interface microstructure of these alloys, as shown in Figure 35 and Figure 36, and have stated that Ti plays a very important role in the brazing mechanism. Table 1 summarizes the conclusions from different research regarding Cu-Sn-Ti active braze alloys.

## 17. Applications

The Cu-Sn-Ti alloy group was employed in producing high strength ageing materials and for abrasive binders. The Cu-Sn-Ti ternary system has a lower melting point and shows good wear properties along with strong interconnection between abrasives because of their outstanding wetting properties. Titanium alloys have been widely used in the manufacturing of biomaterials due to their excellent corrosion resistance, high specific strength, and non-allergenic nature. Due to the high melting point (1670 °C) and poor machinability, other metals are added to obtain combined properties, and hence Cu-Sn-Ti can efficiently replace Cu-Sn in several applications, as mentioned above in the paper by Akshay et al. [75,76].

## 18. Summary and Future Development

The review on cast Cu-Sn and Cu-Sn-Ti alloys was developed by evaluating up-to-date data and its main conclusions are listed.

- Firstly, cast bronze alloys were investigated in detail. The effects of Sn additions on the microstructure and morphology were precisely described.
- Important properties of bronze alloys, such as tensile strength, hardness, conductivity, corrosion, and wear resistance, were also listed above.
- Analysis of cast samples for bronze alloys along with their phase diagrams and phase relations were mentioned.
- Changes in the microstructure and morphology were obtained when different types of casting procedures, such as TZCC, investment casting and continuous casting, were performed. They were explained along with the effect of annealing and rolling.
- Eutectic, hypereutectic and peritectic compositions of bronze alloys were taken into account and their major applications were derived. They included electronic interconnections, underwater equipment (although inferior to stainless steel), detonating cords etc. They are also used in anti-seizure applications.
- Then, the additions of Ti on bronze alloys were taken into account and elaborated using research articles.
- Properties, phase diagrams, and phase relations along with major applications of cast Cu-Sn-Ti alloys were listed.

When comparing both cast alloy groups (Cu-Sn and Cu-Sn-Ti alloys), the cast Cu-Sn-Ti alloys turned out to be efficient replacement alloys for bronze because of their better wear-resistance, but they are not suitable for high load-bearing applications. The high tensile strength of Ti can promote its use as an abrasive material, and for applications where the material is exposed to high-stress levels. Future research can be made on this alloy to confirm their suitability for aerospace and marine industry applications as they show outstanding corrosion resistance, lightweight and tensile properties. Cu-Sn-Ti alloys are also used in biomedical applications.

## Figures and Tables

**Table 1:** Significant conclusions derived from research papers.

Sl.No.	Author and Reference	Materials/Cu-Sn-Ti braze Alloy	Conclusions Derived
1.	Lin et al. [68]	Alumina ceramics	The goal of this work was to investigate the wettability of the Cu/Sn/Ti alloy over polycrystalline alumina. The best wetting ability to alumina substrate is demonstrated by a 70Cu-21Sn-9Ti alloy with 9 wt%Ti brazed at 900 °C. Because Sn is tightly linked with Ti, Sn concentration in Cu/Sn/Ti brazes should be kept below 21% by weight. Three stages could be identified in the brazing filler metal, along with the reaction layer, according to the SEM backscattered electron image (BSE). They were (1) the Cu, Sn, Ti, oxide containing reaction layer; (2) Cu/Sn/Ti intermetallic phase, and the (3) bronze matrix solid solution containing 0.1 wt% Ti.
2.	Li et al. [78]	Between diamond grids and steel substrate	At the temperatures of 925 °C and 1050 °C, diamond grits were brazed onto a steel substrate with a Cu-10Sn-15Ti brazing alloy. In between diamond grits and the braze matrix, a TiC reaction layer was observed. The TiC layer was of grains that were around 50 nm in size, allowing it to efficiently reduce the interfacial stress caused by the diamond and TiC’s lattice mismatch. An intermetallic complex comprised of Sn and Ti was also observed to nucleate and develop into a freely interwoven fine lacey structure on top of the TiC growth front.
3.	Duan et al. [79]	Diamonds, and metal matrix (Fe, Cu, Ni, and Sn)	In this study comparison between different brazing processes was made. Hence, two separate brazing equipment were used: (1) vacuum resistance furnace and (2) high-frequency induction furnace. It was observed that the surface morphology of the pre-brazed diamonds brazing in vacuum resistance furnaces was better. As with the previous studies, TiC intermediate reaction layer was formed indicating any occurrence of chemical combination between Cu–Sn–Ti alloy and the diamond grain. Ti concentration was observed to be higher in the interface of diamond/Cu-Sn-Ti alloy indicating its involvement in the joining process.
4.	Zhang et al. [80]	Diamond and KSC82 carbon steel.	Novel brazing diamond wire saws were performed by applying Cu-Sn-Ti alloy and high-temperature brazing technique to consolidate diamond grits and metallic wire matrix. The findings revealed the presence of a novel Ti2C phase at the interface, where diamond particles were brazed together via reactive wetting. When compared to KSC82 metallic wire, although tensile and yield strength decreased by 41 and 60%, the plasticity increased two-fold, which was said to meet the mechanical performance requirements as stated by the author. The failure due to the separation of diamond grits is caused by two primary reasons. To begin with, the detection of oxygen in the micro-domain of the void lip, as well as the oxidation of Ti, might result in false welding of diamond particles, resulting in early diamond particle separation. Second, the initiation and growth of fatigue crack may cause the diamond grits to lose their holding force at the interface.
5.	Yin et al. [81]	Diamond and Q460 steel substrate	Cu-Sn-Ti composite fillers reinforced with various amounts of tungsten carbide (WC) particles were used to link diamond particles to Q460 steel. The diamond grits had a superior shape and exposure height than the non-WC particle-reinforced samples. Furthermore, including WC particles caused fewer cracks to form at the interface between Cu-Sn-Ti fillers and diamond. The inclusion of 15 wt% WC particles enhanced the formation of TiC and Cu-Ti compounds while preventing the fast intermetallic reaction of Fe and Ti, decreasing the occurrence of brittle phases. As the WC particle concentration increased, the microhardness at the segment interface rose from 179 HV0.05 to 206 HV0.05. The shearing strength of brazed diamond segments reinforced with 15% WC improved by 10%.
6.	Liu et al. [82]	Diamond, c-BN, Al_2_O_3_ and SiC abrasive crystals on 0.45% C steel matrix	Cu-Sn-Ti active powder filler alloys were utilised to braze diamond, CBN, Al_2_O_3_, and SiC abrasive crystals onto a 0.45 percent Carbon steel matrix in a vacuum to construct a new superhard abrasive wheel. The Ti in the Cu-Sn-Ti filler alloy was observed to segregate primarily towards the surface of diamond, CBN, Al_2_O_3_ or SiC, forming a Ti intermediate reaction layer. The Ti-rich layer included phases such as (Ti-C), (Ti-N), (Ti-B), (Ti-O), (Ti-Si), and Ti-(Cu-Al) compounds thus confirming the fact that the chemical metallurgical combination was produced between the grains and the active filler. The metallurgical bonding of active-grain fillers and active filler-steel produced a strong connection between the grains and the 0.45 %C steel substrate. It has been demonstrated that the brazed grains’ reliable bonding strength to the steel substrate could potentially fulfil industrial requirements.
7.	Buhl et al. [83]	Monocrystalline block-shaped diamonds onto a stainless-steel substrate	Three distinct brazing temperatures (880, 930, and 980 °C) and two different dwell periods (10 and 30 min) were used to join monocrystalline block-shaped diamonds with a stainless-steel substrate using a Cu-Sn-based active filler alloy. At the filler-steel intermetallic layer, the following was formed: (1) intermetallic (Fe, Cr, Ni)_2_Ti, and (2) intermetallic phases CuSn_3_Ti, CuSnTi, Ni_2_TiSn and NiTiSn. Residual stresses have a strong relationship with brazing parameters, i.e., either developing compressive (at temperatures of 880 °C and 930 °C) or tensile residual stresses (at temperature 980 °C). Maximum compressive residual stress of around 350 MPa was observed at a holding time of 10 min at 930 °C.
8.	Ding et al. [84]	Between Polycrystalline CBN grains and steel matrix	Cu-Sn-Ti brazing alloy was used to braze polycrystalline CBN grains with AISi 1045 steel matrix. Ti in molten Cu-Sn-Ti brazing alloy interacts with AlN binder and CBN particles of polycrystalline CBN grains forming TiN, TiB_2_, TiB and TiAl_3_ compounds hence proving any evidence of chemical reaction between Cu-Sn-Ti alloy and CBN grains. A strong connection between polycrystalline CBN, steel matrix and Cu-Sn-Ti filler alloy was obtained. The predominant fracture mechanism of brazed polycrystalline CBN grains was the intercrystalline fracture at the CBN-CBN particle boundary.
9.	Fan et al. [85]	CBN	When the brazing temperature is lower than 1223 K, fully uncoated and/or partly coated CBN particles with jagged edges were still observed, and the reaction layer, which is mostly made up of TiN and TiB_2_, appears uneven and thin. When the brazing temperature reaches 1223 K, Ti diffuses completely and is enriched at the interface, resulting in a more homogeneous, continuous, and stable reaction layer composed largely of TiB, TiB_2_ and TiN. Further raising the temperature to 1273 K is unnecessary, if not detrimental, because the reaction layer thickness stays nearly constant and some microscopic microcracks were observed in the interfacial area, reducing the grinding capacity of the final superabrasive product.
10.	Fan et al. [86]	CBN/Cu-Sn-Ti	Through FIB-TEM-EDS-SADP analysis of the interfacial reaction layer at CBN/Cu-Sn-Ti active filler metal at the temperature 1223 K, it was revealed that the interfacial reaction layer is composed mainly of continuous TiB_2_/TiB/TiN layer and irregular TiN/TiB_2_ layer. The reaction layer thickness was observed to be about 1.24 µm. It was proved that metallurgical interfacial bonding was observed, which according to the author can be very useful for the development of high-quality CBN grinding tools.
11.	Hsieh et al. [87]	Graphite	The wetting behaviour of Cu-Sn-Ti brazing alloys on graphite and phase formation at temperatures from 850 °C to 1000 °C was investigated in this study. To promote the wetting of the brazing alloy on graphite, a minimum brazing temperature of 1000 °C was required for Cu–Sn–Ti alloys with Ti concentrations as high as 70 wt%. High amounts of CuSn_3_Ti_5_ and SnTi_3_ intermetallic compounds were observed, with an increase in Ti concentration and a reduction in Sn concentration. In a ductile Sn-rich matrix phase, however, a rise in Sn concentration and a reduction in Ti concentration might result in the precipitation of intermetallic compounds such as Sn_3_Ti_5_ and Sn_3_Ti_2_. The optimum Ti and Sn concentrations for effective wetting on graphite at low temperatures, while retaining a significant volume fraction of ductile phases, were around 10 wt% Ti and 15 wt% Sn.

## Data Availability

The data presented in this study are available on request the corresponding author.
